# Efficient synthesis of some [1,3]-oxazine derivatives in the presence of solid acid nano catalyst based on ferrierite and study on their activity against breast cancer through molecular docking calculation

**DOI:** 10.1038/s41598-024-67292-3

**Published:** 2024-07-13

**Authors:** Atiyeh khollat, Leila Moradi

**Affiliations:** Department of Organic Chemistry, Faculty of Chemistry, University of Kashan, P.O. Box 8731753153, Kashan, Iran

**Keywords:** [1,3]-oxazine, Magnetic solid acid catalyst, Ferrierite, Green chemistry, M-FER/SO_3_H, Chemical biology, Computational biology and bioinformatics

## Abstract

In this research, the magnetic solid acid nanocatalyst based on ferrierite has been prepared and used as catalyst for the green synthesis of some [1,3]-oxazine derivatives in water at room temperature. The synthesized compounds were obtained in high to excellent yields after short reaction times and the structure of synthesized products were investigated by spectroscopic methods such as: FT-IR, ^1^H NMR and ^13^C NMR. The prepared magnetic solid acid catalyst was characterized using XRD, FT-IR, FE-SEM, EDX, elemental mapping, TGA and VSM analysis methods. Magnetic catalyst has easy separation ability, which leads to better and easier recycling. The preparation and synthesis of [1,3]-oxazine derivatives were carried out at room temperature in the presence of M-FER/TEPA/SO_3_H. Easy workup, green solvent (water) and also short reaction times with high to excellent yield of products, are some of advantageous of presented method. Docking calculations on the structure of the synthesized compounds proved their medicinal properties against breast cancer cells.

## Introduction

Multicomponent reactions (MCRs) as green, eco-friendly, with atom, time and energy economy are applicable routs to efficient synthesis of heterocyclic compounds^1–4^. Multicomponent synthesis of [1,3]-oxazine heterocycles have attracted a lot of attention due to their medicinal properties such as anti-cancer, anti-pain, anti-fungal, anti-tuberculosis, anti-bacterial, anti-blood pressure and anti-thrombosis^5,6^. Burke et al. were reported the synthesis of oxazines in 1952 through the condensation of β-naphthol with aniline derivatives and formaldehyde^5^. Shinde et al. were prepared [1,3]-oxazine derivatives in PEG-400 media as a safe condition^6^. Alum was used as a non-toxic and recyclable catalyst by Sadaphal et al. for the synthesis of [1,3]-oxazine compounds^7^. Kategaonkar performed the synthesis of [1,3]-oxazine by various anilines derivatives, 1-naphthol and formaldehyde using ZrOCl_2_ catalyst at room temperature^8^. Dhakane et al. reported a convenient and efficient synthesis of [1,3]-oxazine derivatives using thiamine hydrochloride (VB1)^9^. A synthetic method for the preparation of [1,3]-oxazine derivatives was reported by Borah et al. The high yield products were obtained at room temperature in ethanol solvent using Cl_3_CCOOH^10^. Babaei et al. reported the synthesis of [1,3]-oxazine derivatives by Al_2_O_3_/BF_3_/Fe_3_O_4_ nanocatalyst at room temperature^11^. Some of reported acidic catalysts have shown good results for the synthesis of [1,3]-Oxazines, but unfortunately, these catalysts are homogeneous and toxic. Solid acid catalysts as heterogeneous, eco-friendly and recyclable catalysts, do not produce acidic wastes^12–15^. The immobilization of sulfonic acid on different types of solid supports, leads to the production of new heterogeneous solid acid catalysts as an environmentally friendly approach in synthetic chemistry^16–21^.

**Scheme 1.**
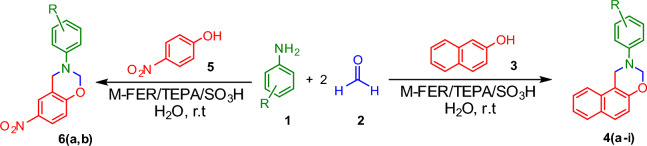
Preparation of [1,3]-Oxazine derivatives in the presence of M-FER/TEPA/SO_3_H.

Zeolites as microporous alumino-silicate materials commonly used as commercial acid adsorbents, catalyst and also catalyst support^22^. Zeolites can be synthesized with different chemical compositions and distinct frameworks^23^. Ferrierite as rare natural mineral, is divided into three similar main groups: Mg-ferrierite, Na-ferrierite and K-ferrierite, which is named based on the dominant cation in the structure of ferrierite. Mg-ferrierite and K-ferrierite are orthorhombic minerals and Na-ferrierite is monoclinic ferrierite with very different cation composition. Silica ferrierite is a zeolite with medium pores and a very high percentage of silica. Ferrierites are commonly found as metamorphic minerals in basaltic rocks^24,25^. In recent years, ferrierite was used as catalyst and catalyst support for the preparation of efficient and reusable catalysts^22,26, 27^. Also, magnetization of catalysts gives them a good separation ability under an external magnetic field. In recent years, the application of magnetic catalysts attracted most attention of chemists^28–30^. In this research, the synthesis of [1,3]-oxazine derivatives have been carried out in the presence of M-FER/SO_3_H magnetic nano catalyst based on the principles of green chemistry (Scheme 1). Computational investigation of docking on the prepared structure was performed by Schrödinger 2020 software (Maestro 12/5). It is possible to evaluate the conformation of the three-dimensional structure of a potential drug on its receptor. If the ligand docks well with the receptor and locks into the receptor, it is possible that it can be effective in terms of biological activity. Docking calculation, enables a medicinal chemist to better assess the structure between drugs and receptor with it. If the ligand binds well with the dock receptor and inside the receptor, it is possible to have biological activities^31^.

## Experimental

### Materials and methods

Aniline derivatives, p-nitrophenol, 2-naphthol, formaldehyde, FeCl_3_,6H_2_O, FeCl_2_,4H_2_O, ammonia, tetra ethylene pentamine, epichlorohydrin and chlorosulfonic acid were obtained from Merck and Aldrich company. The surface morphology of catalyst was characterized by FESEM instrument (Mira3-XMU). The crystalline structure of catalyst was obtained with Philips X’pert MPD diffractometer. FT-IR spectra were recorded using PerkinElmer 781 spectrophotometer and KBr pellets to determine the functional groups in the range of 400–4000 cm^−1^. For more confirmation of [1,3]-oxazines structure, Bruker ^1^H NMR and ^13^C NMR on DRX-400 spectrometer were applied. Thermal weighing analysis was obtained with the DuPont 2000 TGA V5.1A device.

### Synthesis of catalyst

#### Synthesis of magnetic Ferrierite nanoparticles (M-FER)

**Scheme 2.**
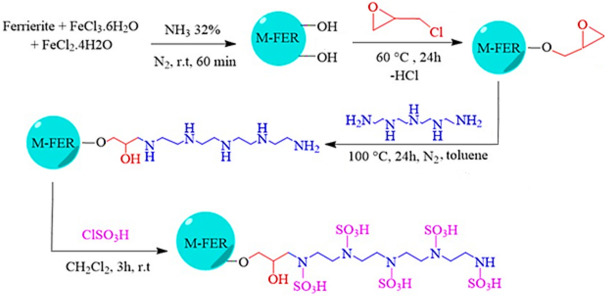
The preparation of magnetic solid acid nanocatalyst based on ferrierite (M-FER/TEPA/SO_3_H).

1 g of ferrierite was poured into a 50 ml round bottom flask and 1.5 g of FeCl_3_. 6H_2_O_,_ 0.75 g of FeCl_2_. 4H_2_O, and 10 ml of deionized water was added. Then, ammonia (32%) was added dropwise under N_2_ atmosphere until black precipitate was formed and stabilized on the ferrierite surfaces. The obtained mixture was stirred for 1 h at room temperature and then, separated with a strong magnet, washed with DI water and dried in at 80 °C (Scheme 2).

#### Modification of magnetic Ferrierite nanoparticles by epichlorohydrin linker (M-FER/ECH)

1 g of M-FER, 2.27 ml of epichlorohydrin and 2 ml of ethanol were added to a 50 ml flask and stirred at 60 °C for 24 h by a magnetic stirrer. Afterwards, the mixture was centrifuged to separate the M-FER/ECH. Then, the nanoparticles were washed twice with ethanol and at the end, was dried at 80 °C (Scheme 2).

#### Modification of M-FER/ECH by tetra ethylene pentamine (M-FER/ECH/*TEPA*)

To attach TEPA on M-FER/ECH nanoparticles, 0.7 g of the modified substrate from the previous step (M-FER/ECH), along with 20 ml of toluene were poured into a 50 ml flask placed in an ultrasonic bath for 10 min. Then, 1 ml of TEPA was added and the reaction was performed at 100 °C for 24 h under nitrogen atmosphere. After the time, functionalized nanoparticles were separated by an external magnetic field and washed twice with ethanol and finally dried at 80°C (Scheme 2).

#### Preparation of Solid acid catalyst nanoparticles based on Ferrierite M-FER/ECH/TEPA/SO_3_H

To prepare the solid acid catalyst, 0.5 g of the FER/ECH/TEPA obtained from the previous step, with 10 ml of dichloromethane was added to a 50 ml flask and dispersed in the ultrasonic bath for 20 min. After that, 1.5 ml of chlorosulfonic acid was added dropwise to the reaction mixture during 20 min under a laboratory fume hood and then, stirred for 3 h at room temperature using a magnetic stirrer. At the end, the produced nanoparticles were separated by a magnet and washed with dichloromethane and ethanol and dried (Scheme 2). The acidic site of solid acid catalyst was determined by back titration method. According to obtained results, the concentration of catalytic sites (H^+^) was 0.95 mmol.g^-1^.

#### Synthesis of [1,3]-oxazine derivatives in the presence of (M-FER/TEPA/SO_3_H)

A mixture of β-naphthol/p-nitrophenol (1 mmol), formaldehyde (2 mmol) and aniline derivatives (1 mmol) were added to 2 ml of distilled water and mixed at room temperature in the presence of 0.015 g of the prepared catalyst (the reaction progress was monitored by TLC during all stages of the reaction). After completion of the reaction, the catalyst was separated with strong magnet, washed with ethanol and water and dried for other reactions. The pure product was obtained by filtration of reaction mixture and washed with ethanol and water and dried at 80° (Scheme 1).

### Representative spectral data

#### 2-(4-chlorophenyl)-3,2-dihydro-1H-naphtho[1,2-e] [1,3]-oxazine (4a)

FTIR (KBr) ῡ (cm^−1^): 3437, 2915, 2857, 1624, 1491, 1394, 1225, 1002, 1181, 812; ^1^H NMR (400 MHz, CDCL_3_, δ, ppm): 4.95 (s,2H, Ar-CH_2_-N), 5.42 (s,2H, O-CH_2_-N), 7.05–7.12 (m, 4H, Ar-H), 7.23 (d, *J* = 8.9 Hz, 1H, Ar-H), 7.41 (t, *J* = 8.1 Hz, 1H, Ar-H), 7.55 (t, *J* = 7.5 Hz, 1H, Ar-H), 7.69 (t, *J* = 7.7 Hz, 2H, Ar-H), 7.80 (d, *J* = 7.8 Hz, 1H, Ar-H).

#### 2-(4-Bromophenyl)-3,2-dihydro-1H-naphtho[1,2-e] [1,3]-oxazine (4b)

FT-IR (KBr) ῡ (cm^−1^): 3425, 1629, 1483, 1390, 1223, 1001, 1180, 810; ^1^H NMR (400 MHz, CDCL_3_, δ, ppm): 4.95 (s,2H, Ar-CH_2_-N), 5.42 (s,2H, O-CH_2_-N), 7.05 (d, *J* = 9.0 Hz, 2H, Ar-H), 7.39–7.43 (m, 4H, Ar-H), 7.55 (t, *J* = 8.3 Hz, 1H, Ar-H), 7.69 (t, *J* = 8.7 Hz, 2H, Ar-H), 7.80 (d, *J* = 8.2 Hz, 1H, Ar-H).

#### 2-(4-Iodophenyl)-3,2-dihydro-1H-naphtho[1,2-e] [1,3]-oxazine (4c)

FT-IR (KBr) ῡ (cm^−1^): 3428, 3257, 2924, 2858, 1626, 1479, 1398, 1237, 1117, 810; ^1^H NMR (400 MHz, CDCl_3_ δ ppm): 5.01 (s,2H, Ar-CH_2_-N), 5.35 (s,2H, O-CH_2_-N), 6.66 (d, *J* = 8.7 Hz, 4H, Ar-H), 7.15 (d, *J* = 8.8 Hz, 2H, Ar-H), 7.80 (t, *J* = 6.9 Hz, 2H, Ar-H), 7.82 (d, *J* = 8.2 Hz, 1H, Ar-H), 7.90 (d, *J* = 8.6 Hz, 1H, Ar-H); ^13^C NMR (100 MHz, CDCl_3_ δ ppm): 30.68, 37.48, 114.93, 114.96, 115.01, 118.08, 122.46, 123.12, 126.37, 128.14, 128.16, 129.02, 133.56, 136.98, 148.42, 153.09.

#### 2-(3-Chloro-4- Fluorophenyl)-3,2-dihydro-1H-naphtho[1,2-e] [1,3]-oxazine (4d)

FT-IR (KBr) ῡ (cm^−1^): 3440, 2950, 2897, 1602, 1498, 1395, 1227, 1002, 1147, 947, 814; ^1^H NMR (400 MHz, CDCl_3_ δ ppm):4.93 (s,2H Ar-CH_2_-N), 5.37 (s,2H O-CH_2_-N), 7.00–7.09 (m, 3H, Ar-H), 7.23 (d, *J* = 7.7 Hz, 1H, Ar-H), 7.42 (t, *J* = 7.0 Hz, 1H, Ar-H), 7.55 (t, *J* = 7.0 Hz, 1H, Ar-H), 7.7 (dd, *J* = 8.7,3.4 Hz, 2H, Ar-H), 7.82 (d, *J* = 8.1 Hz, 1H, Ar-H); ^13^C NMR (100 MHz, CDCl_3_ δ ppm): 30.42, 48.35, 79.05, 111.38, 115.37, 116.15, 116.41, 118.19, 120.27, 120.56, 123.39, 126.40, 128.08, 128.28, 128.63, 130.62, 145.27, 151.45.

#### 2-(4- Methoxyphenyl)-3,2-dihydro-1H-naphtho[1,2-e] [1,3]-oxazine (4e)

FT-IR (KBr) ῡ (cm^−1^): 3333, 3055, 2925, 2862, 1588, 1510, 1456, 1387, 1241, 1181, 811; ^1^H NMR (400 MHz, CDCl_3_ δ ppm): 3.77 (s,3H, CH_3_), 4.92 (s,2H, Ar-CH_2_-N), 5.38 (s,2H, O-CH_2_-N), 6.83 (d, *J* = 9.1 Hz, 2H, Ar-H), 7.07 (d, *J* = 8.9, 1H, Ar-H), 7.13 (d, *J* = 9.0, 2H, Ar-H), 7.40 (t, *J* = 6.9 Hz, 1H, Ar-H), 7.53 (t, *J* = 7.0 Hz, 1H, Ar-H), 7.69 (t, *J* = 8.5 Hz, 2H, Ar-H), 1.02 (d, *J* = 7.5 Hz, 1H, Ar-H).

#### 2-(4- Nitrophenyl)-3,2-dihydro-1H-naphtho[1,2-e] [1,3]-oxazine (4f)

FT-IR (KBr) ῡ (cm^−1^): 3431, 2921, 1600, 1478, 1394, 1321, 1265, 1010, 1191; ^1^H NMR (400 MHz, CDCl_3_ δ ppm): 5.09 (s,2H, Ar-CH_2_-N), 5.53 (s,2H, O-CH_2_-N), 7.12 (d, *J* = 8.9 Hz, 1H, Ar-H), 7.18 (d, *J* = 9.3 Hz, 2H, Ar-H), 7.45 (t, *J* = 7.6 Hz, 1H, Ar-H), 7.60 (t, *J* = 7.1 Hz, 1H, Ar-H), 7.73 (t, *J* = 8.5 Hz, 2H, Ar-H), 7.84 (d, *J* = 8.1 Hz, 1H, Ar-H), 8.22 (d, *J* = 9.4 Hz, 2H, Ar-H).

#### 2-(3- Nitrophenyl)-3,2-dihydro-1H-naphtho[1,2-e] [1,3]-oxazine (4g)

FT-IR (KBr) ῡ (cm^−1^): 3429, 3085, 3015, 2894, 1592, 1493, 1380, 1349, 1230, 1105, 1008; ^1^H NMR (400 MHz, CDCl_3_ δ ppm): 5.06 (s,2H, Ar-CH_2_-N), 5.51 (s,2H, O-CH_2_-N), 7.08 (d, *J* = 8.9 Hz, 1H, Ar-H), 7.52–7.39 (m, 3H, Ar-H), 7.57 (t, *J* = 7.8 Hz, 1H, Ar-H), 7.85–7.66 (m, 4H, Ar-H), 8.04 (s, 1H, Ar-H).

#### 2-(2- Nitrophenyl)-3,2-dihydro-1H-naphtho[1,2-e] [1,3]-oxazine (4h)

FT-IR (KBr) ῡ (cm^−1^): 1621, 1575, 1510, 1430, 1390, 1341, 1274, 1232, 1157; ^1^H NMR (400 MHz, CDCl_3_ δ ppm): 4.48 (s, 2H, Ar-CH_2_-N), 5.04 (s, 2H, O-CH_2_-N), 6.71 (t, *J* = 7.6 Hz, 1H, Ar-H), 6.82–6.86 (m, 4H, Ar-H), 7.03 (d, *J* = 8.5 Hz, 2H, Ar-H), 7.23 (d, *J* = 8.6 Hz, 1H), 7.38 (t, *J* = 7.6 Hz, 1H, Ar-H), 7.54 (t,* J* = 8.5 Hz, 1H, Ar-H).

#### 2-(2,4- Dinitrophenyl)-3,2-dihydro-1H-naphtho[1,2-e] [1,3]-oxazine (4i)

FT-IR (KBr) ῡ (cm^−1^): 1629, 1500, 1423, 1333, 1263, 919, 1122; ^1^H NMR (400 MHz, CDCl_3_ δ ppm): 6.22–6.88 (m, 4H, 2CH_2_), 6.93 (d, *J* = 9.2 Hz, 2H, Ar-H), 7.28 (d, *J* = 3.6 Hz, 3H, Ar-H), 8.25 (dd, *J* = 9.2, 2.6 Hz, 2H, Ar-H), 9.14 (d, *J* = 2.6 Hz, 2H, Ar-H); ^13^C NMR (125 MHz, CDCl_3_ δ ppm): 38.76, 39.60, 118.05, 119.41, 119.70, 122.98, 123.27, 127.98, 128.31, 128.59, 128.89, 129.18, 134.77, 135.06, 149.49, 149.75, 151.40, 151.69.

#### 3-(3-Chloro-4-methylphenyl)-6-nitro-3-4-dihydro-2H-benzo[e] [1,3]-oxazine (6a)

FT-IR (KBr) ῡ (cm^−1^): 3434, 1599, 1499, 1403, 1346, 1227, 1144, 941, 802; ^1^H NMR (400 MHz, DMSO-*d*_*6*_ δ ppm): 4.41 (s,2H, Ar-CH_2_-N), 4.92 (s,2H, O-CH_2_-N), 6.59–6.62 (m, 2H, Ar-H), 6.80 (s, 1H, Ar-H), 7.11 (t, *J* = 9.1Hz, 2H, Ar-H), 7.26 (d,* J* = 9.0 Hz, 1H, Ar-H); ^13^C NMR (125 MHz, DMSO*-d*_*6*_ δ ppm). 69.43, 76.78, 116.25, 116.47, 117.74, 117.97, 119.99, 120.18, 120.63, 120.82, 144.83, 144.78, 151.62, 154.06.

#### 3-(4-Boromophenyl)-6-nitro-3-4-dihydro-2H-benzo[e] [1,3]-oxazine (6b)

FT-IR (KBr) ῡ (cm^−1^): 3429, 3039, 2929, 2846, 1592, 1493, 1380, 1330, 1226, 1162, 809; ^1^H NMR (400 MHz, CDCl_3_ δ ppm): 4.79 (s,2H, Ar-CH_2_-N), 5.09 (s,2H, O-CH_2_-O), 6.69 (dd, *J* = 6.3Hz, 2.3 Hz, 2H, Ar-H), 6.99 (d, *J* = 5.2 Hz, 2H, Ar-H), 7.16 (d, *J* = 9.1 Hz, 2H, Ar-H), 7.33 (s, 1H, Ar-H).

## Results and discussion

The Morphology, composition and elemental distribution of the ferrierite nanoparticles and M-FER/TEPA/SO_3_H was investigated by FE-SEM imaging, EDX and elemental mapping techniques.

**Figure 1:**
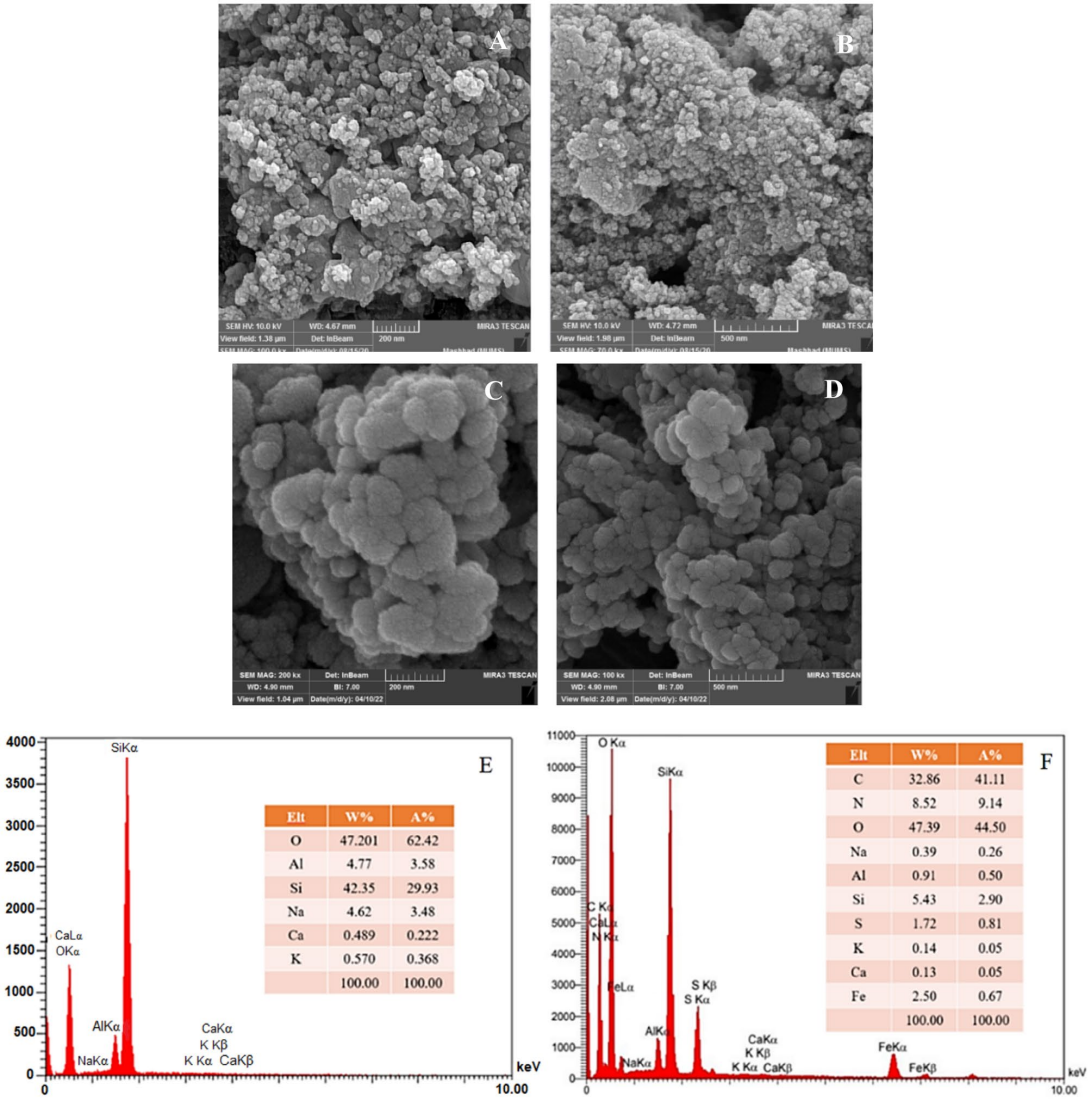
FE-SEM of ferrierite (**A**, **B**), M-FER/TEPA/SO_3_H (**C**, **D**), and EDX of ferrierite (**E**) and M-FER/TEPA/SO_3_H (**F**).

Figure 1A, B indicated clearly that the raw ferrierite nanoparticles have spherical morphology. Moreover, the spherical morphology was not changed in M-FER/TEPA/SO_3_H; yet, little changes are observed in the surfaces, which indicates the functionalization with sulfonic acid groups Fig. 1C, D.

**Figure 2:**
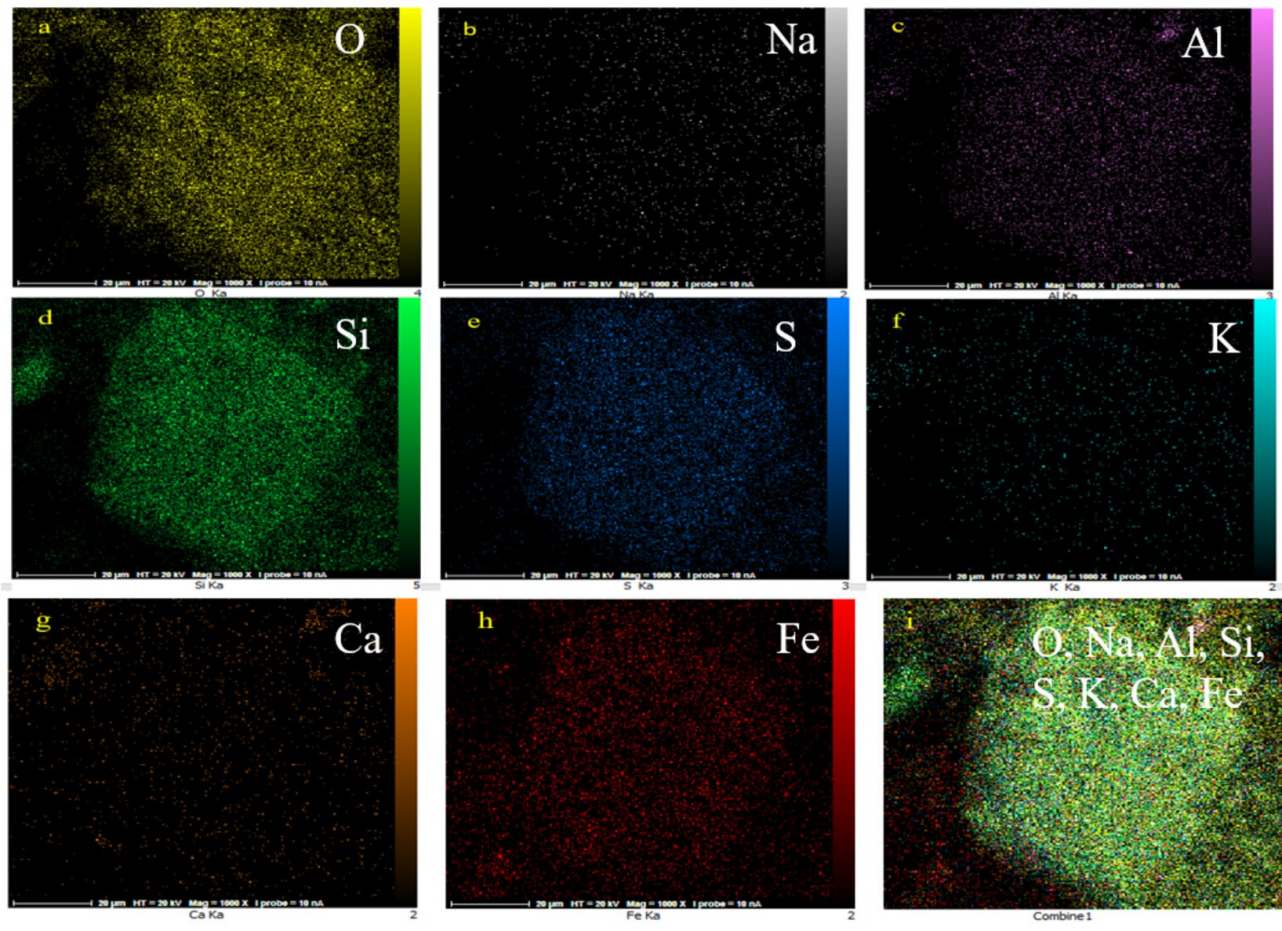
Elemental mapping of M-FER/TEPA/SO_3_H.

The composition of ferrierite and M-FER/TEPA/SO_3_H was determine using EDX analysis. As can be seen in Fig. 1E, F, the presence of main elements like Si, Al, O and also Na, Ca and K in ferrierite structure was confirmed. Also, Fe, C, N and S elements in the composition of M-FER/TEPA/SO_3_H demonstrated the successfully synthesis of magnetic solid acid catalyst. Also, the elemental mapping of M-FER/TEPA/SO_3_H confirmed the good dispersion of Fe, O, S and other elements in catalyst structure (Fig. 2).

**Figure 3:**
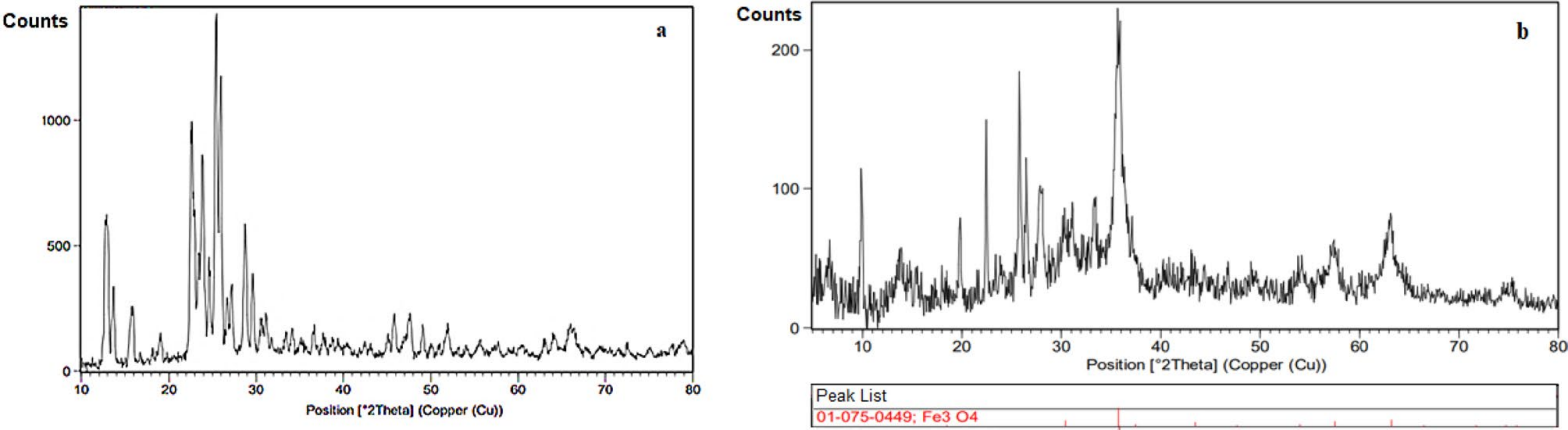
XRD pattern of ferrierite (**a**) and M-FER/TEPA/SO_3_H (**b**).

The XRD pattern of ferrierite and M-FER/TEPA/SO_3_H are shown in Fig. 3. As can be seen, the diffraction peaks correspond to the ferrierite structure (Fig. 3a), is according to the references with JCPDS Card No. 98-009-7919^32–34^. Incorporation of Fe_3_O_4_ NPs and TEPA/SO_3_H on ferrierite surfaces, cause to some changes in the XRD pattern of ferrierite NPs. Moreover, addition to peaks belong to ferrierite NPs, the reflections at 2*θ* = 31.22°, 35.60°, 37.08°, 44.69°, 53.36°, 56.10° and 62.79° contributed to the (220), (311), (222), (400), (422), (511), (440) reflections (JCPDS Card No. 01-075-0449), are related to the magnetic Fe_3_O_4_ nanoparticles stabilized on the ferrierite surfaces.

**Figure 4:**
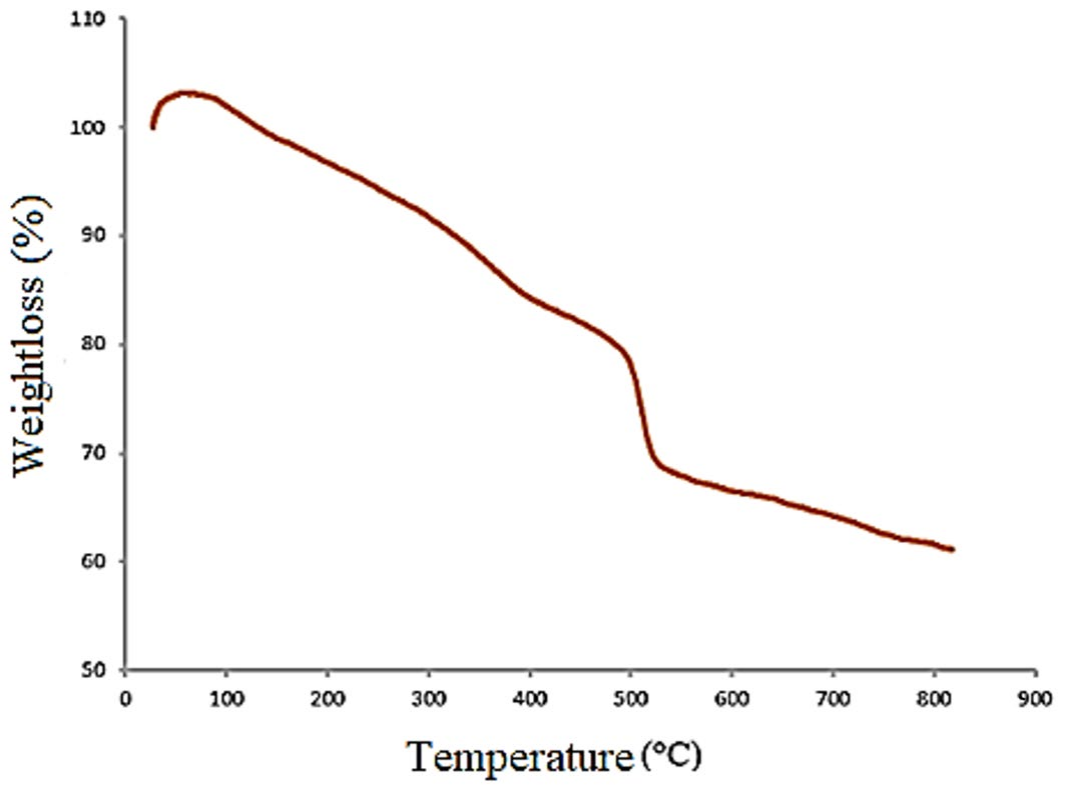
Thermogravimetric analysis of M-FER/TEPA/SO_3_H.

Thermogravimetric analysis of M-FER/TEPA/SO_3_H was done to evaluate the catalyst thermal stability and the percentage of organic groups attached on the M-FER surfaces (Fig. 4). As can be seen, the weight loss (about 3%) occurred in the region of 100–200 °C, is related to the removal of remained solvents between the nano catalyst particles. In addition, a further weight loss (17%) in the region of 200–500 °C indicates the separation of the amino sulfate groups attached to the M-FER surfaces and a sudden weight loss (12%) at 500 °C is form the removal of ECH attached to the M-FER surfaces. Finally, low decrease in weight (7%) occurs in the region of 530–800 °C, is from the separation of hydroxyl groups.

**Figure 5:**
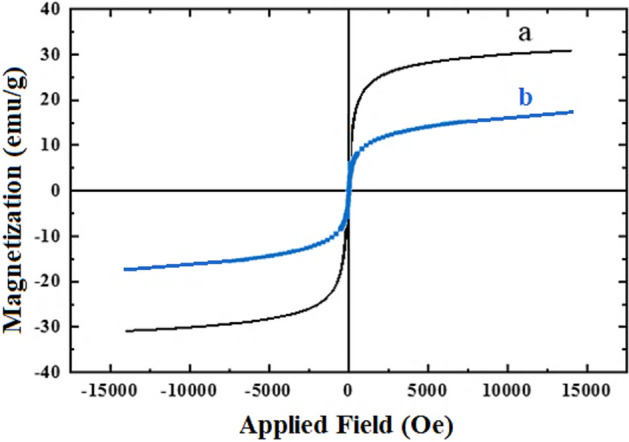
VSM of Fe_3_O_4_ nanoparticles (**a**) and M-FER/TEPA/SO_3_H (**b**).

To Evaluate of magnetic feature of prepared catalyst, VSM analysis was done. As can be seen in Fig. 5, the magnetic behavior of catalyst is clearly confirmed and shows the magnetization about 20 emu/g, which is lower than raw Fe_3_O_4_ nanoparticles with 34 emu/g.

### Optimization of reaction conditions

To investigate the optimum reaction conditions including catalyst amounts, solvent and temperature, reaction of 4-chloro aniline, formaldehyde and β-Naphthol was used as model reaction. To find the best solvent, the model reaction was done in different solvents at room temperature. According to the obtained results (Table 1), water cause to higher yield than other solvents (entry 8). Moreover, the reaction in solvent-free conditions had lower yields (entries 1–4). To the find of best amount of catalyst, the model reaction was performed in the presence of different amounts of catalyst at room temperature. When 0.015 g of catalyst was used in water at room temperature, the product was obtained with the highest yield. Also, the efficiency of the reaction did not improve when catalyst was used higher than the optimal quantity (entry 8). So, based on the results in the Table 1 (entry 7), it can be concluded that the best conditions were 0.015 g of catalyst in the water at room temperature.

**Table 1:** Optimization of reaction conditions^a^.


Entry	Catalyst (g)	Solvent	T (^o^C)	Time (min)	Yield (%)^b^
1	0.015	MeOH	25	3	60
2	0.015	CDCl_3_	25	3	35
3	0.015	CH_2_Cl_2_	25	3	30
4	0.015	–	80	8	75
5	0.015	EtOH	25	3	80
6	0.015	H_2_O	25	3	97
7	0.02	H_2_O	25	3	97

^a^Reaction conditions: 4-chloro aniline (1 mmol), formaldehyde (2 mmol), β-Naphthol (1 mmol) and 2 mL of solvent, TON = 67.8

^b^Isolated yields.

After determining the optimal reaction conditions, [1,3]-Oxazine derivatives were synthesized using various anilines, formaldehyde, 4-nitrophenol or β-naphthol in the presence of 0.015 g of M-FER/TEPA/SO_3_H in H_2_O at room temperature (Table 2). As can be seen, all of products were obtained in high to excellent yields and characterized by ^1^HNMR, ^13^CNMR and FT-IR techniques.

**Table 2:** Synthesis of some [1,3]-oxazines in the presence of M-FER/TEPA/SO_3_H.


Ref	M.P (°C)	Yield (%)	Time (min)	R	Product	Entry
^14^	101–103	97	3	4-Cl	5a	1
^14^	118–121	95	4	4-Br	5b	2
–	144–146	91	6	4-I	5c	3
–	166–168	89	3	3-Cl-4-F	5d	4
^14^	77–79	98	9	4-OMe	5e	5
^6^	168–170	96	7	4-NO_2_	5f.	6
^7^	131–133	95	5	3-NO_2_	5g	7
^7^	111–112	94	12	2-NO_2_	5h	8
**–**	170–172	96	15	2–4-NO_2_	5i	9
**–**	163–165	90	4	3-Cl-4-F	6a	10
**–**	116–118	89	17	4-Br	6b	11

Reaction conditions: aniline derivatives (1 mmol), p-nitrophenol/β-naphthol (1 mmol), formaldehyde (2 mmol) in 5 mL H_2_O in the presence of 0.015 g M-FER/TEPA/SO_3_H at room temperature.

### Proposed mechanism

**Scheme 3.**
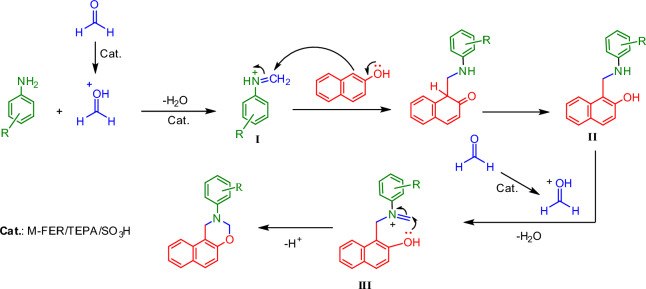
The mechanism for the prepration of [1,3]-oxazines in the presence of M-FER/TEPA/SO_3_H.

The proposed mechanism for the synthesis of [1,3]-oxazines is presented in Scheme 3. At the beginning of the reaction, the first molecule of formaldehyde is activated with solid acid catalyst and then, nucleophilic attack of aniline, (follow by removal of H_2_O), produces the iminium ion (**I**) in the presence of solid acid catalyst ^10^. In continue, from the reaction of intermediate **I** and β-naphthol, intermediate **II** is formed. At the next step, intermediate **II** reacts with the second molecule of formaldehyde in the presence of solid acid catalyst and intermediate **III** is created. Finally, with the intramolecular cyclization of intermediate **III** and removal of H^+^, the final product is formed.

A study on the efficiency of M-FER/TEPA/SO_3_H compare with other reported results for the synthesis of compound **4a** was done and results were collected in Table 3. As shown in Table 3, M-FER/TEPA/SO_3_H shows the best yield after short reaction time (entry 9). This good performance is related to the significant surface area of M-FER/TEPA/SO_3_H with a great deal of acidic sites.

**Table 3:** Comparison of M-FER/TEPA/SO_3_H performance with another reported results^a^.


Entry	Reaction conditions	T (^o^C)	Time (min)	Yield (%)^b^	Ref
1	Nano-Al_2_O_3_/BF_3_/Fe_3_O_4_, H_2_O	25	15	85	^11^
2	Gum arabic-OPO_3_H_2_, solvent-free	25	9	97	^35^
3	SiO_2_-BF_3_, sonication, solvent-free	25	15	89	^36^
4	SiO_2_-BF_3_, solvent-free	25	60	70	^36^
5	Fe_3_O_4_@walnut shell/Cu(II), solvent-free	60	15	89	^37^
6	Without catalyst, H_2_O	25	30	82	^38^
7	Fe_3_O_4_@nano-cellulose/TiCl, solvent-free	25	3	96	^14^
8	FNAOSiPAMP/Cu(II)	25	25	87	^39^
9	M-FER/TEPA/SO_3_H, H_2_O	25	3	97	–

### 1ERE protein

1ERE proteins are the α estrogen receptors (ERα) belong to the nuclear receptor superfamily. They are receptors that were activated by the estrogen hormone (17β-estradiol)^40^. Estrogen receptors (ER) are over-expressed in around 70% of breast cancer cases and referred to as "ER-positive”. Available evidence suggests that two mechanisms are exist in this case: (a) binding of estrogen to the ER which cause to stimulates proliferation of mammary cells that increase the cell division and DNA replication and lead to mutations and (b) Generation of genotoxic wastes from estrogen metabolism. The result of both processes is disruption of cell cycle, apoptosis and DNA repair, which increases the chance of tumor formation^41^.

### Examining how 1ERE binds to its natural ligand (estradiol)

Estradiol (E2) as a steroid hormone, is a key regulator of growth, differentiation and physiological functions in a wide number of target tissues including the male and female reproductive system, neuronal, skeletal and cardiovascular systems. The predominating mechanisms of estrogen action are mediated through binding to the nuclear estrogen receptor (ER), which induces transcription of target genes containing estrogen response element (ERE)^42^. The ER is part of a large nuclear receptor superfamily that shares common structure and function/domains. This receptor family acts as the signal transmitter for most of the known fat-soluble hormones, including steroids (androgen receptor, (AR); progesterone receptor, glucocorticoid receptor, mineralocorticoid receptor), retinoids, thyroid hormones and vitamin D^43^.

**Figure 6:**
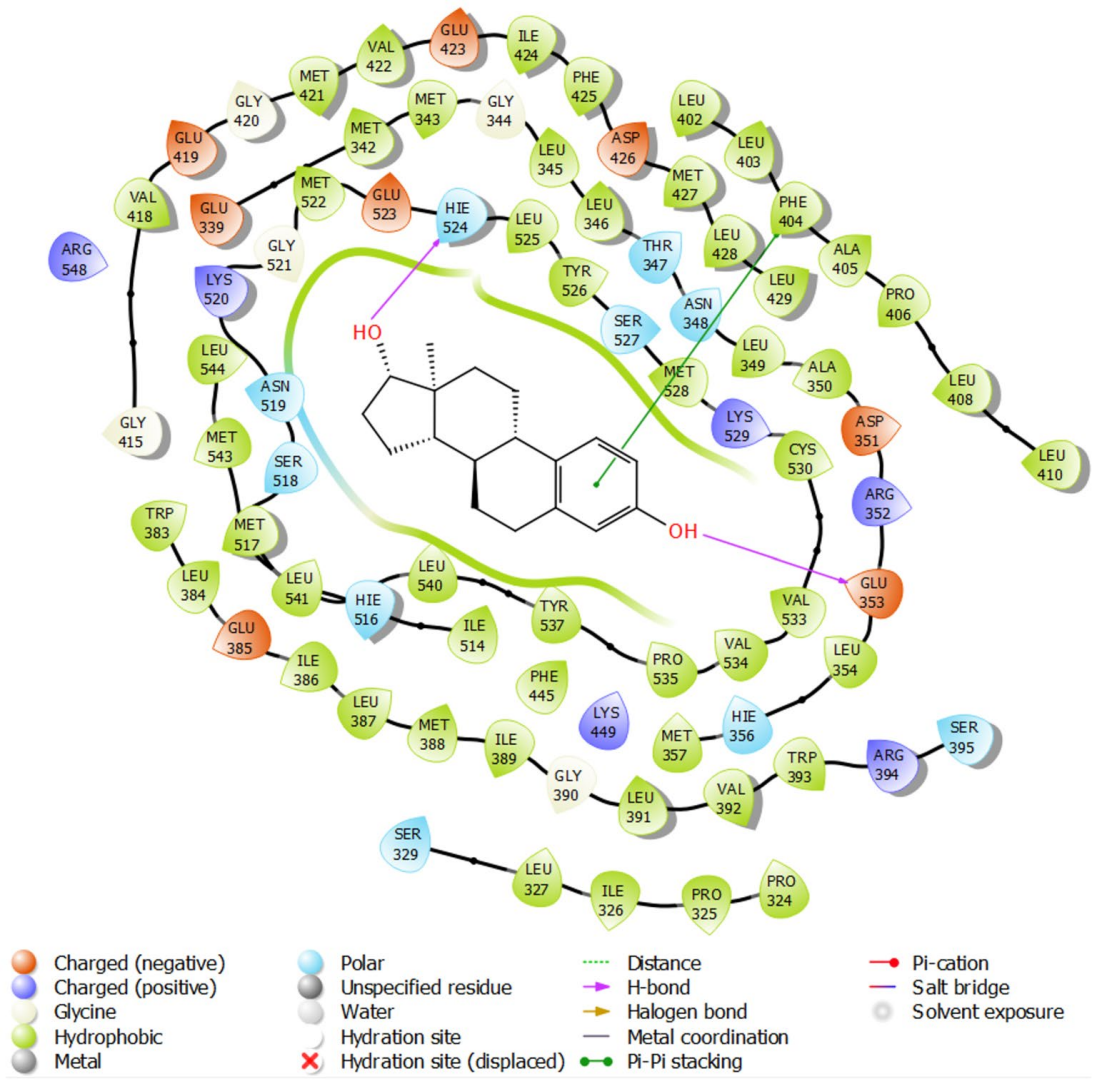
1ERE protein binds to its main ligand estradiol.

Binding of estradiol ligand (as agonist and main ligand) with 1ERE protein is shown in in Fig. 6. Two hydrogen bonds with histidine residue 524 (due to its polar NH groups) and glutamic acid 353 (due to its negative charge of COO^-^) and the non-covalent π-π interaction between the aromatic ring of estradiol and the aromatic ring of phenylalanine residue 404 can be observed. These linkages have a very special and vital role in biological and pharmaceutical communication^44,45^.

#### Performing molecular docking calculations of [1,3]-oxazine derivatives on 1ere protein

The docking results of the synthesized [1,3]-oxazine compounds are shown in Table 4. The Glide Score or docking energy indicates the binding strength between the ligand and its receptor. The low amounts of docking energy illustrate a stronger binding. The results according to Lee Pinsky's rules^46^ are shown in the Table 4 as follow:

1. Molecular weight (MW): In this rule, the molecule weight of the drug should not be more than 500 g/mol, because as the molecule becomes heavier, its absorption and permeability will be decrease. All synthesized compounds follow this rule.
2. Predicted octanol / water partition coefficient (QLogP0/w): This rule tries to establish a balance between hydrophilicity and lipophilicity of the drug molecule. In this item, the octanol/water partition coefficient should not be more than 5. This applies to 7 compounds, but 4 compounds 4a, 4b, 4c, 4d are too lipophile, but this problem can be easily solved by adding a hydrophilic molecule such as sugar molecules to the compound, or the drug can be administered intravenously without any changes.
3. The number of hydrogen donor groups (H-Bond donator): This rule indicates the number of hydrogen donor groups in the drug molecule. Groups such as NH and OH and their number should not be more than 5. All synthesized compounds follow this rule.
4. Number of hydrogen acceptor groups (H-Bond acceptor): This item shows the number of hydrogen acceptor groups. These groups are O and N and their number should not be more than 10. All synthesized compounds also follow this rule.
5. None of these compounds is a Transporter Substrate.

**Table 4:** Molecular docking results of [1,3]-oxazine derivatives on 1ere protein.

Entry	MW	Gllide score	PHOA^a^	QlogPs	QPPCaco	H-bond donor	H-bond acceptor	QPlogP_0_/W
4h	306.320	− 8.314	100	− 4.428	1654.833	0	2.750	3.904
4a	295.768	− 7.937	100	− 5.556	9679.423	0	1.750	5.088
4d	313.75	− 7.930	100	− 5.810	9641.998	0	1.750	5.271
4g	306.320	− 7.848	100	− 4.540	1156.841	0	2.750	3.824
4e	219.349	− 7.749	100	− 4.695	9676.430	0	2.50	4.626
4c	378.219	− 7.738	100	− 5.821	9687.477	0	1.750	5.256
6a	308.696	− 7.251	100	− 4.616	1095.413	0	2.750	3.573
4b	340.219	− 7.139	100	− 5.687	9678.784	0	1.750	5.167
4f.	306.320	− 7.052	100	− 4.545	1153.775	0	2.750	3.820
6b	335.156	− 6.995	100	− 4.527	1097.250	0	2.750	3.448

^a^PHOA = Prediction of human oral absorption on a scale of 0–100 percent. Recommended values: > 80% is high and < 25% is poor.

Predicted apparent Caco-2 cell permeability in nm/sec (QPPCaco): This item plays an important role in bioavailability and drug absorption. Cell permeability optimizes the gastrointestinal absorption of drugs, Recommended values < 25 are poor and > 500 are great. All of synthesized compounds have QPPCaco over than 500.

Predicted aqueous solubility (QLogPS): This item also plays a very important role in gastrointestinal absorption and oral bioavailability of the drug. Recommended values are -6.5 to + 0.5. according to the results, all of compounds have acceptable amounts of aqueous solubility.

#### Schematic illustration of binding of [1,3]-oxazine compounds on 1ERE protein

**Figure 7:**
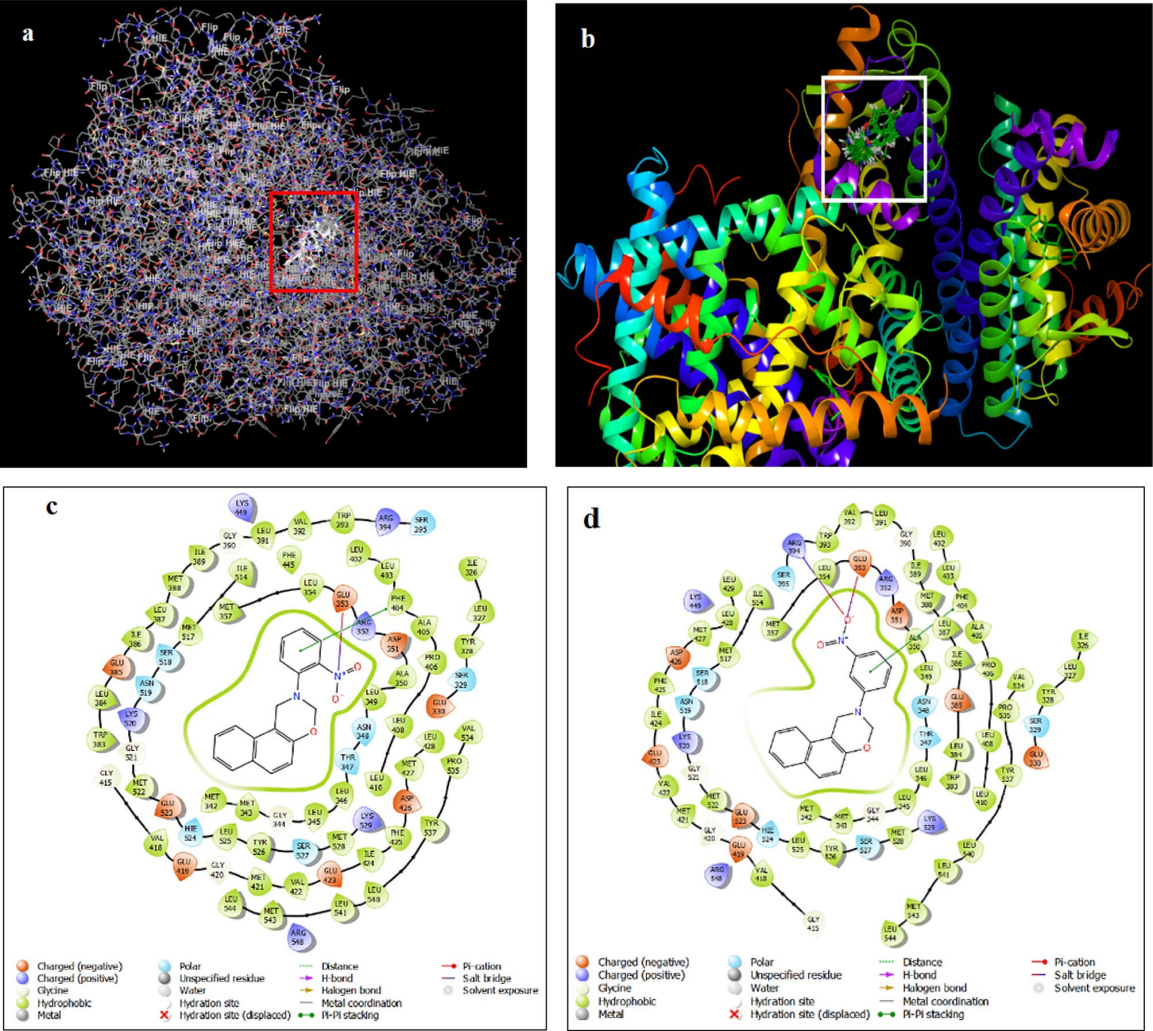
Molecular docking of [1,3]-oxazine derivatives on 1ERE protein ligand (**a**, **b**), and the interaction of **5g** and **5h** with protein (**c**, **d**).

As shown in Fig. 7a, b, all of compounds bind to the active site of the 1ERE protein, (or so-called docked). These connections cause the inactivation of this protein and create favorable effects in the treatment of breast cancer. In fact, estradiol agonist ligand will not be able to activate this protein in the body. Additionally, the binding interacting of compounds **5g** and **5h** whit protein is shown in Fig. 7c, d. These connections are as the same as known estrogen antagonist compounds^44,45^. In this research, we tried to synthesis antagonist compounds structurally similar to the active antagonists. Also, it has been tried to make these compounds significantly smaller and more applicable which leads to better interaction with ER receptors. This claim has been confirmed with the molecular docking calculations (Table 4).

### Catalyst leaching

To investigate the heterogeneous nature of Perlite M-FER/TEPA/SO_3_H, the leaching test has been carried out. Initially, a mixture of 4-chloro aniline (1 mmol), formaldehyde (2 mmol), β-Naphthol (1 mmol) and 0.015 g of M-FER/TEPA/SO_3_H in H_2_O (2 mL) was provided and stirred for 90 s at room temperature. After the time, the catalyst was separated with a magnetic field. The process was monitored by TLC and the yield of product was about 47%. After that, the reaction mixture was stirred for another 90 s at room temperature. After the time, the reaction efficiency was checked (which was 47%) and no improvement was shown in the reaction. obtained results indicated that the catalyst has heterogeneous behavior and the progress of the reaction is depended on the use of the solid acidic catalyst.

### Catalyst reusability

**Figure 8:**
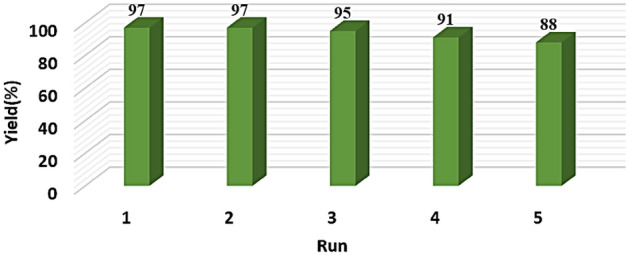
Reusability of M-FER/TEPA/SO_3_H.

The convenient separation and reusability of the catalyst has always been considered as a factor to determine the efficiency of catalysts. Heterogeneous catalysts have these advantageous over homogeneous catalysts. To this purpose, after the completion of model reaction, the magnetic nanocatalyst was separated by a strong magnet and washed repeatedly by ethanol and distilled water, dried at 80 °C and used for subsequent reactions. Catalyst recycling was done for 5 steps and as can be seen in Fig. 8, no considerable changes was occurred in the catalyst efficiency (Supplementry material).

## Conclusion

In this research, magnetic ferrierite nanoparticles were prepared and modified with sulfamic acid groups (M-FER/TEPA/SO_3_H) as an efficient solid acid catalyst. The magnetic property of the designed catalyst led to its easy recycling. The synthesis of [1,3]-oxazine derivatives were carried out under green conditions (at room temperature in water as green solvent) in a short period of time and products were obtained in high to excellent yields. Moreover, molecular docking calculations shown that, the synthesized compounds have the potential to become as an anti-breast cancer drugs.

### Supplementary Information

Supplementary Figures.

## Data Availability

All of the data generated or analyzed during this study, can be found in the published article and its supplementary information file.
