# Supplementary material for: Efficient synthesis of some [1,3]-oxazine derivatives in the presence of solid acid nano catalyst based on ferrierite and study on their activity against breast cancer through molecular docking calculation
Source: Sci Rep. 2024 Jul 13;14:16211. doi: 10.1038/s41598-024-67292-3 (PMC11246498; doi:10.1038/s41598-024-67292-3)
Supplement: Supplementary file 1 — Supplementary Figures. [file 41598_2024_67292_MOESM1_ESM.pdf]

Green synthesis of some [1,3]- oxazine derivatives in the presence of solid acid nano catalyst based on ferrierite and study on their computational molecular docking

Atiyeh khollat and Leila Moradi

Department of Organic Chemistry, Faculty of Chemistry, University of Kashan, P.O. Box 8731753153, Kashan, Iran. E-mail: l_moradi@kashanu.ac.ir

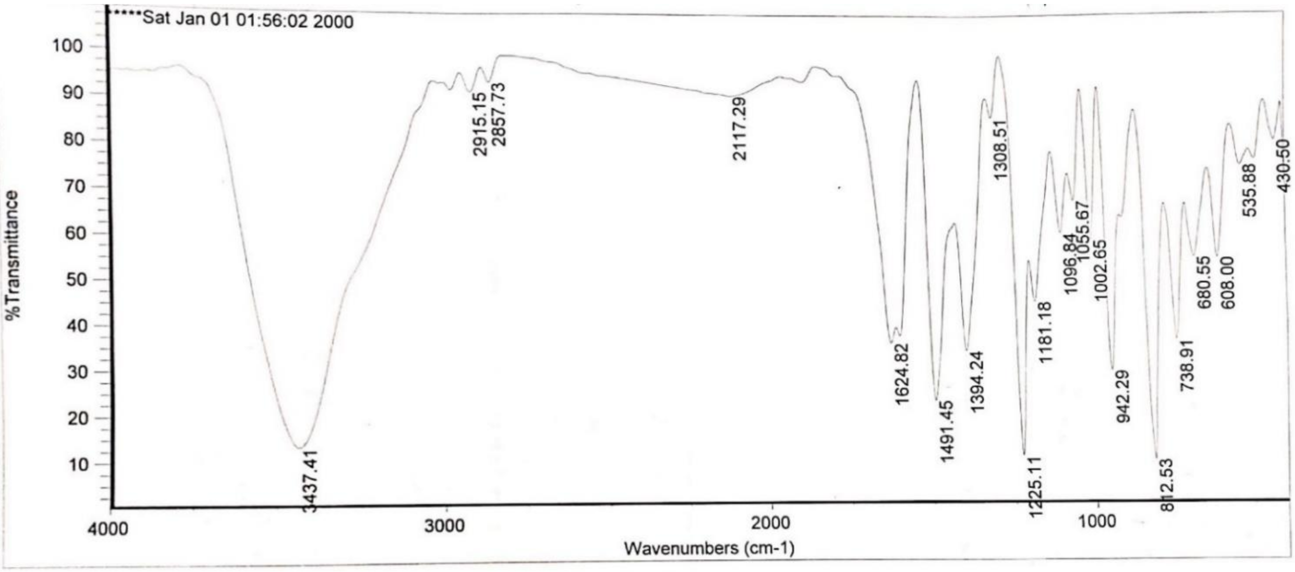

FT-IR of 4a

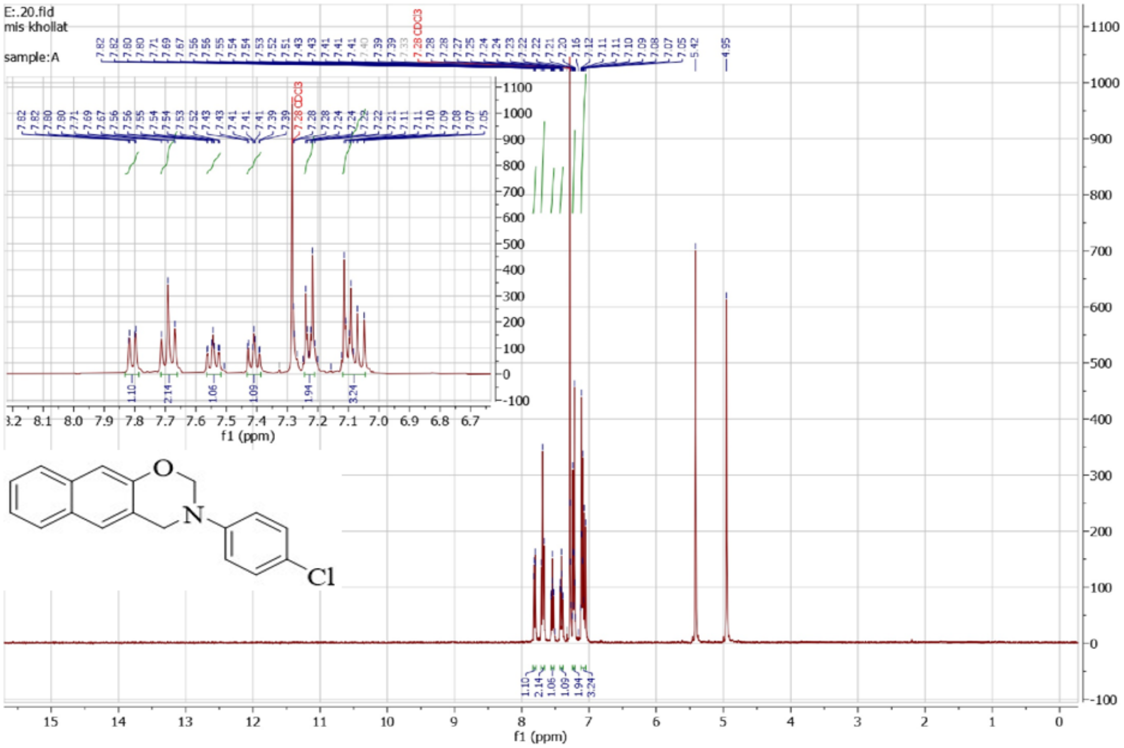

Figure 2.^{1}H NMR 4a

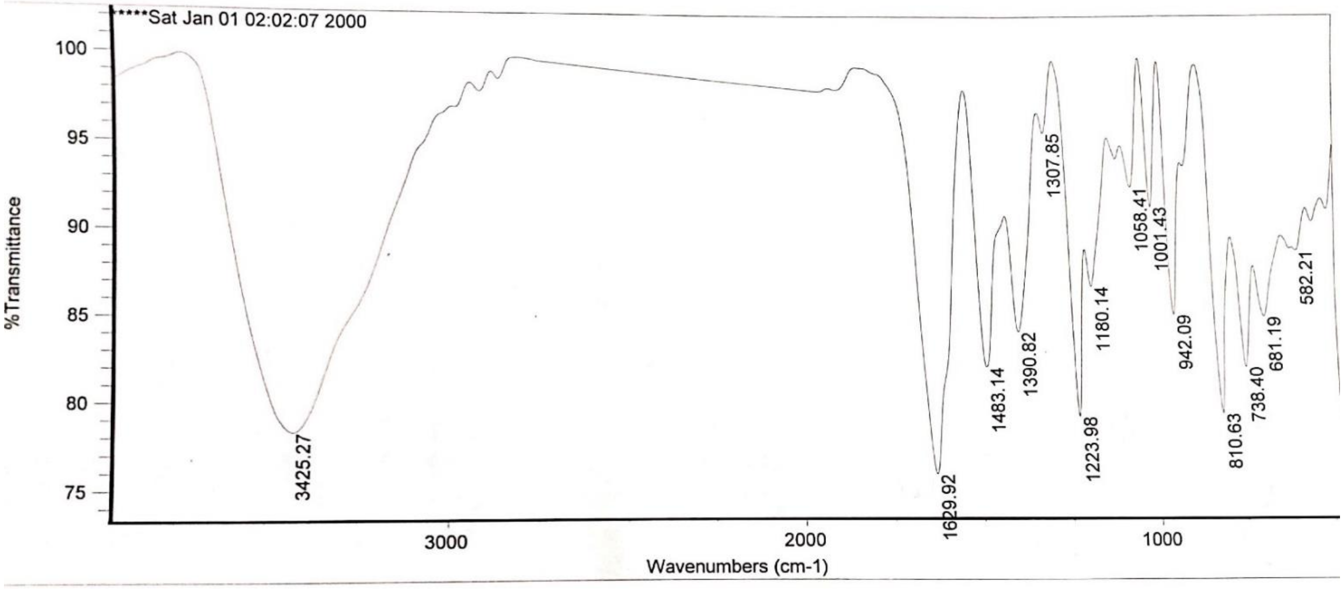

FT-IR of 4b

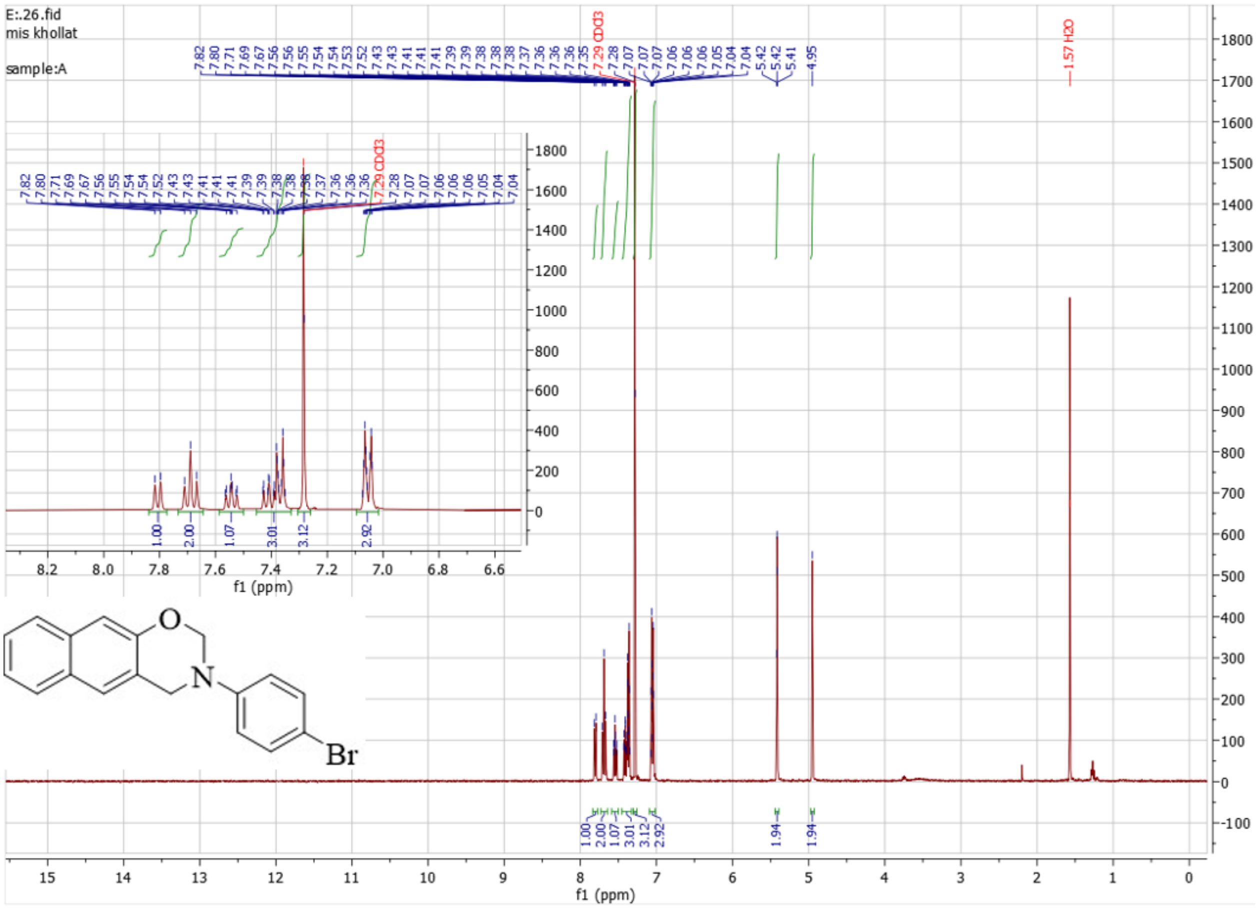

^{1}H NMR of 4b

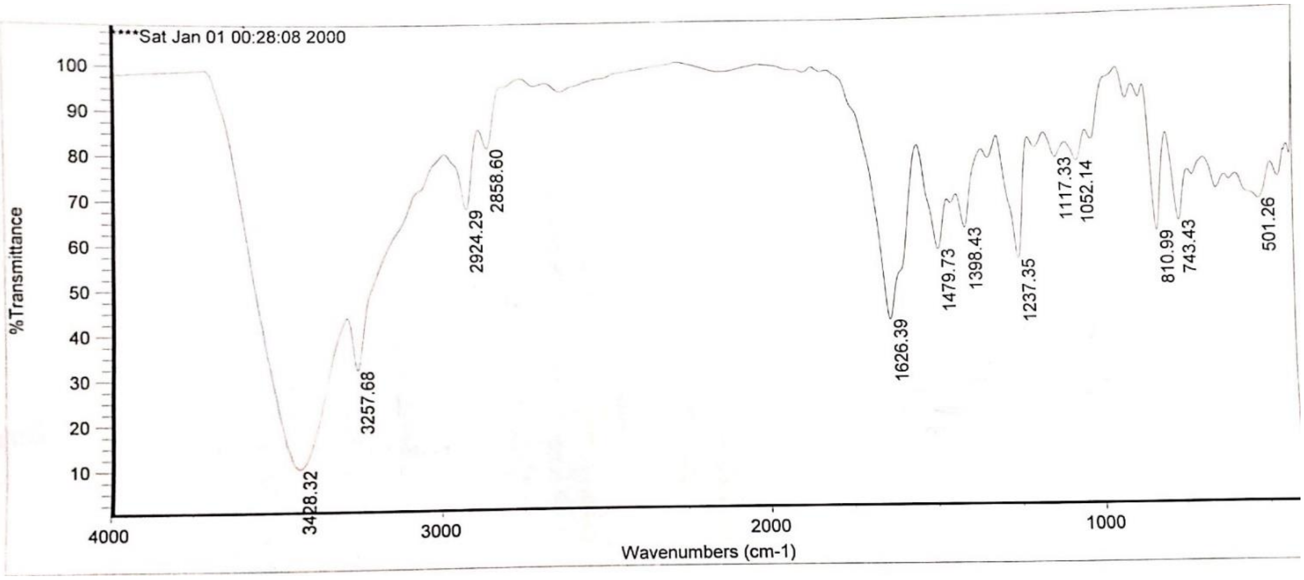

FT-IR of 4c

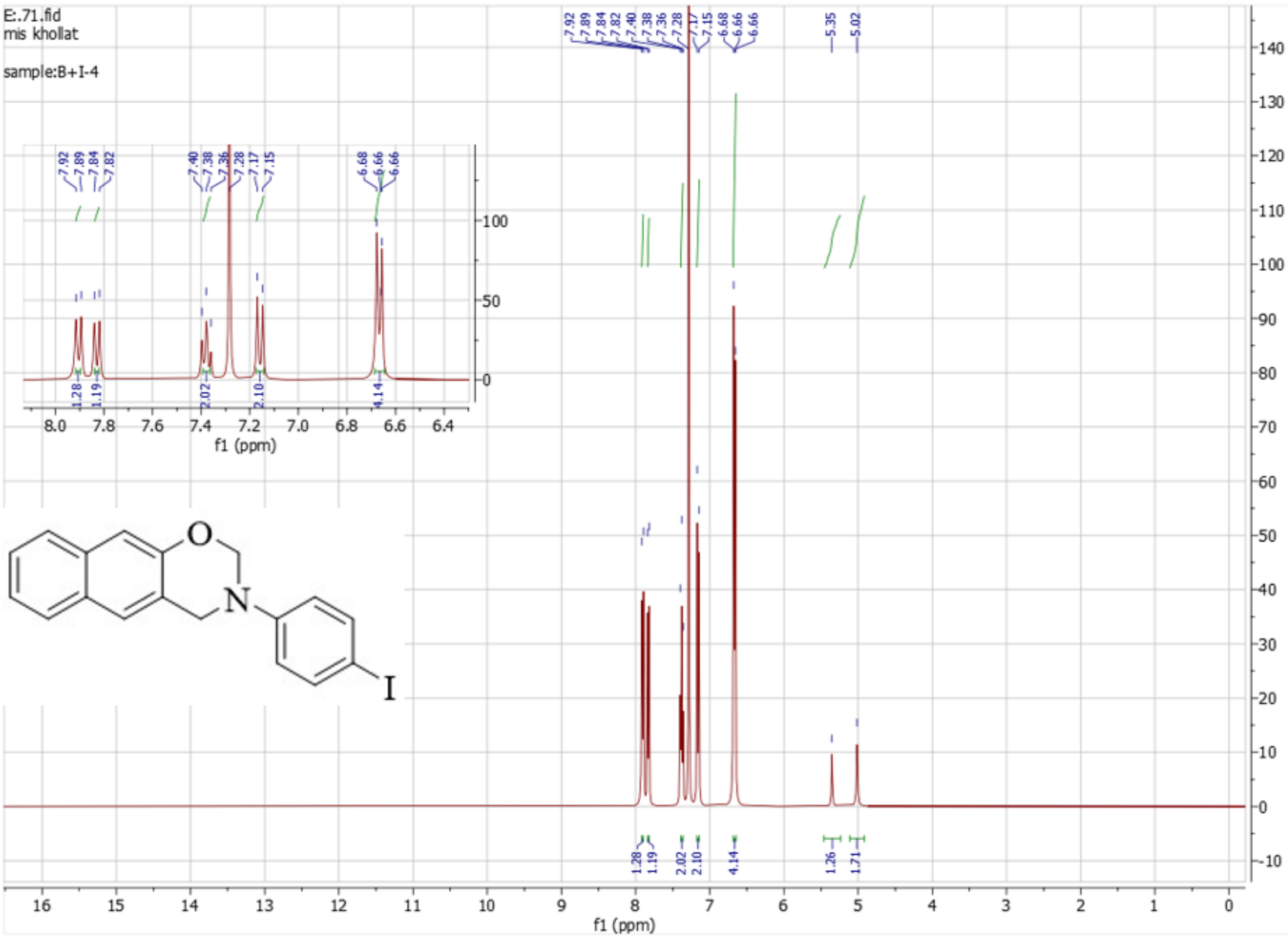

^{1}H NMR of 4c

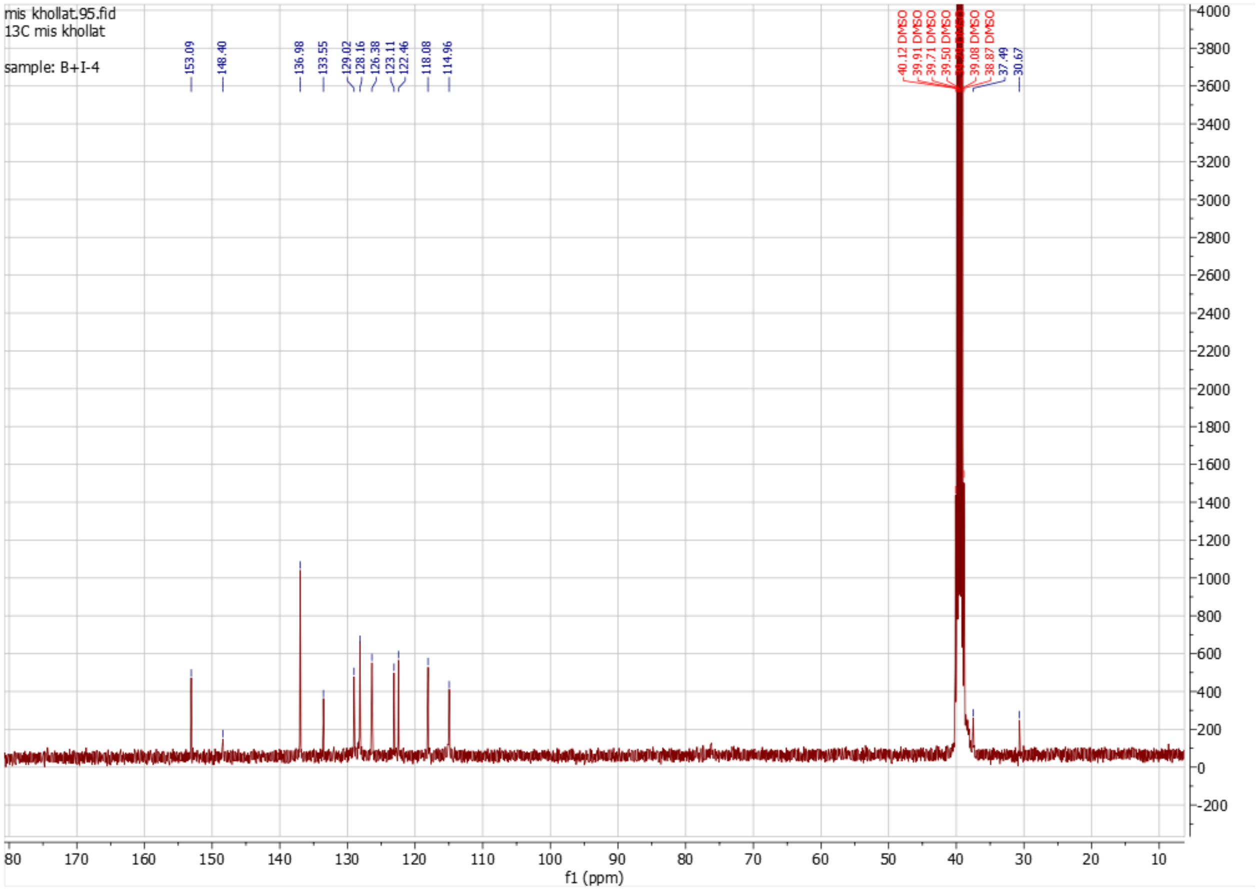

^{13}C NMR of 4c

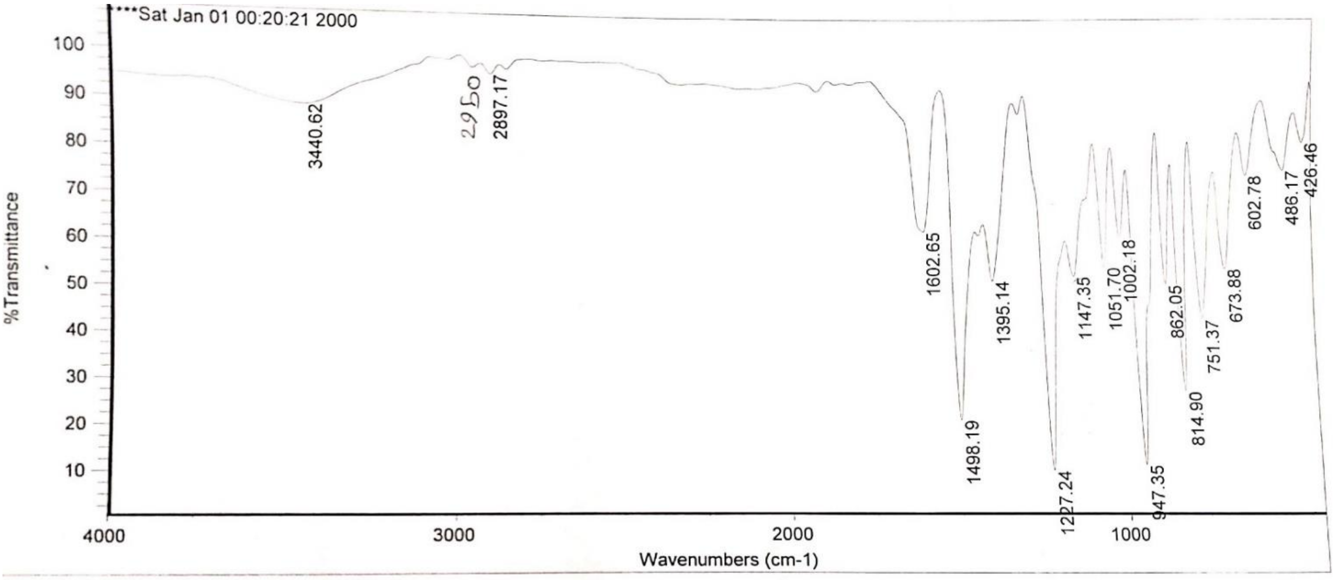

FT-IR of 4d

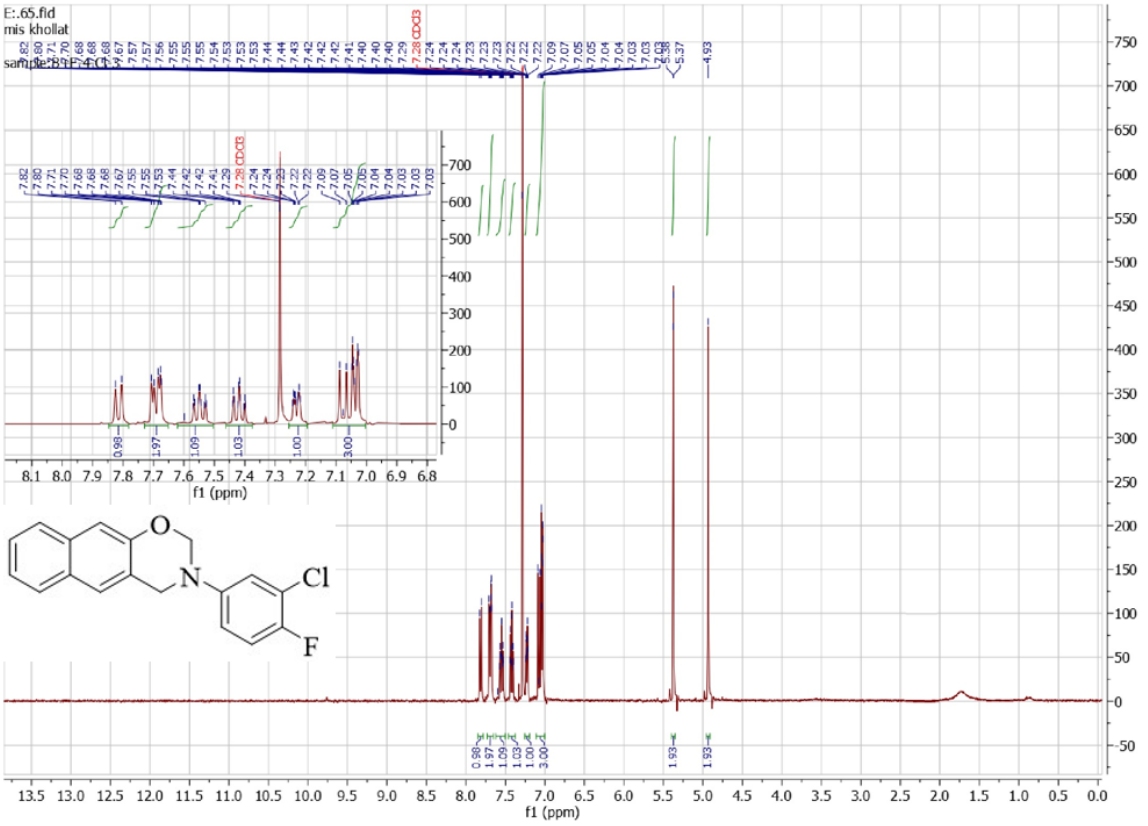

^{1}H NMR of 4d

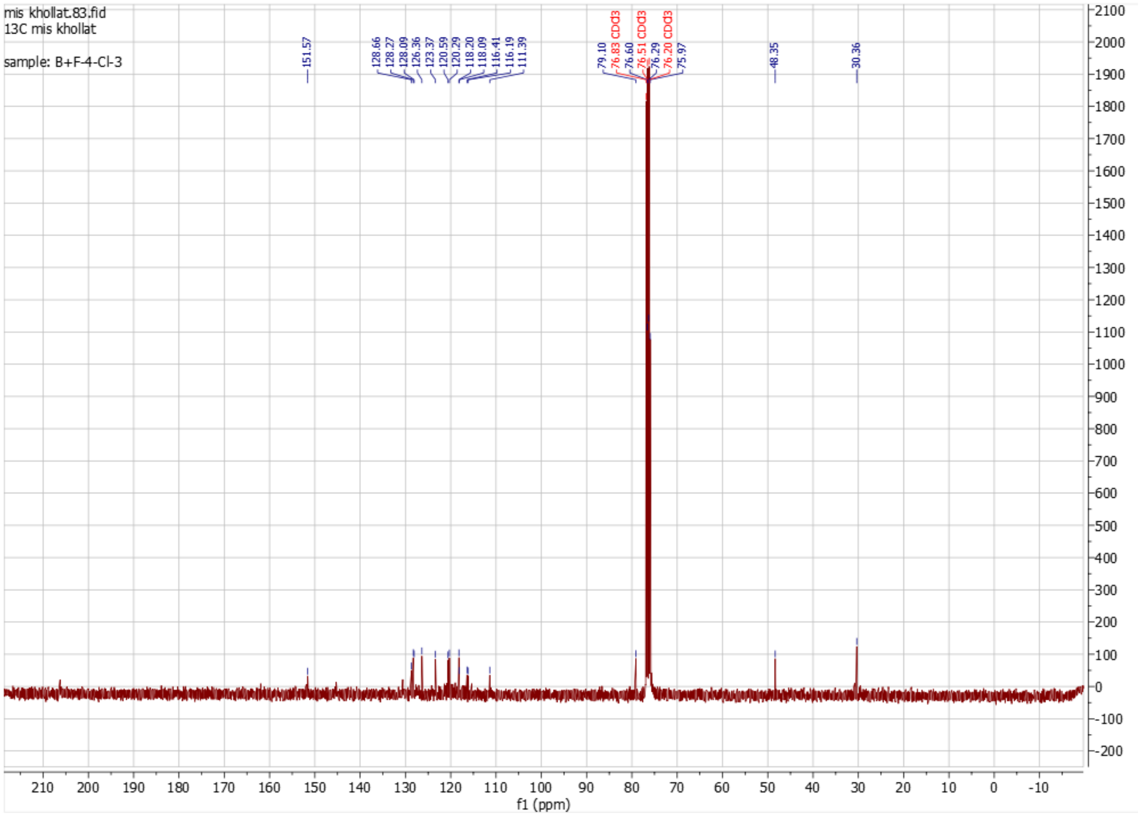

^{13}C NMR of 4d

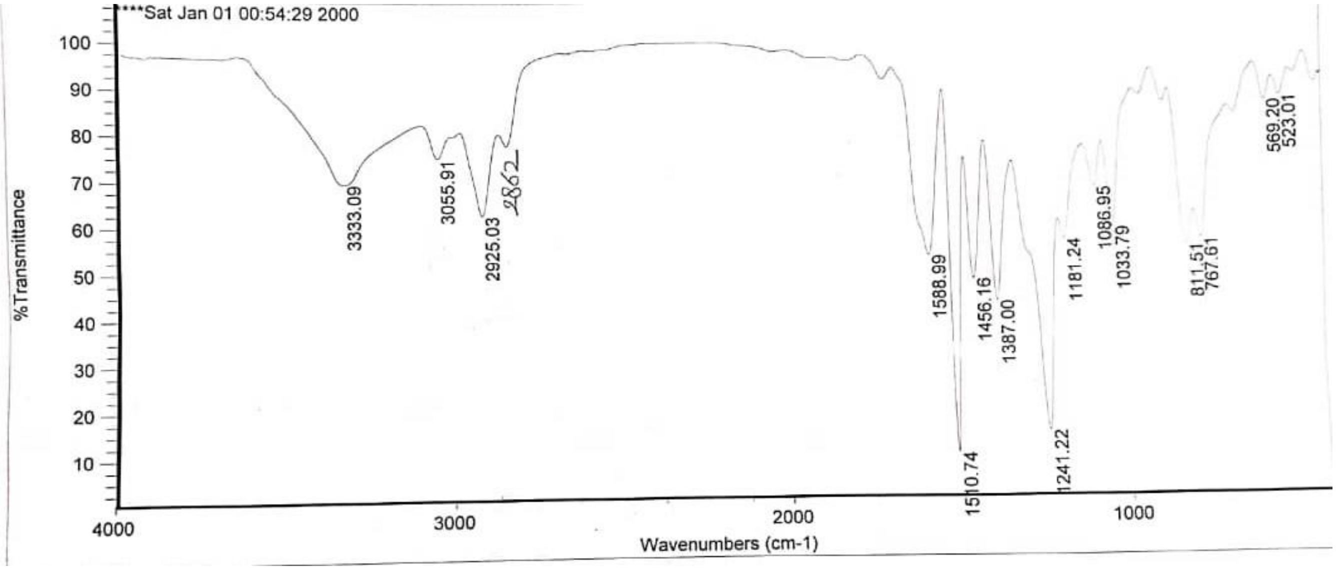

FT-IR of 4e

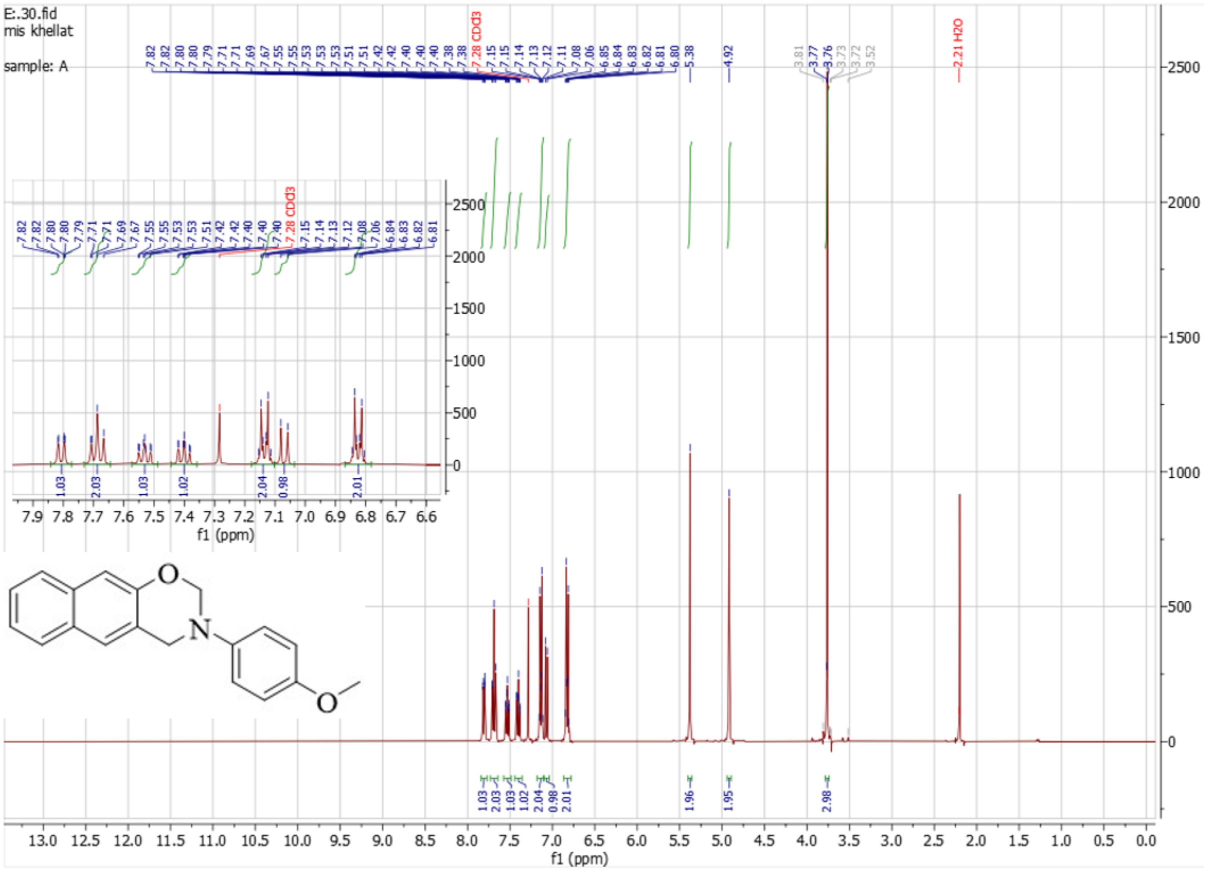

^{1}H NMR of 4e

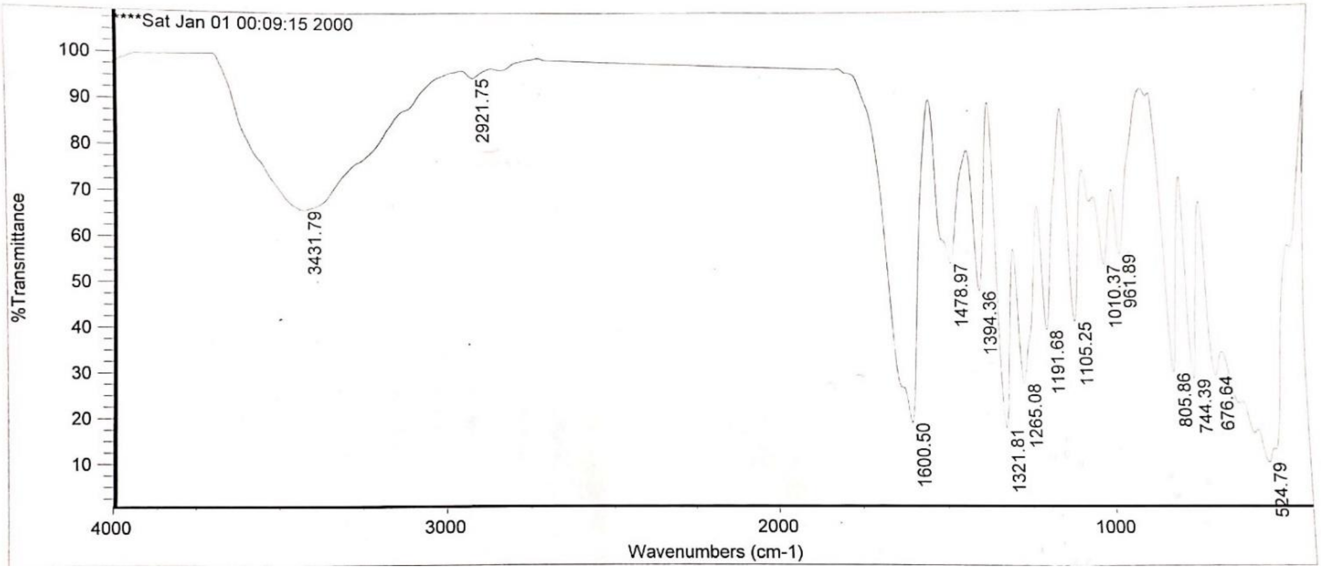

FT-IR of 4f

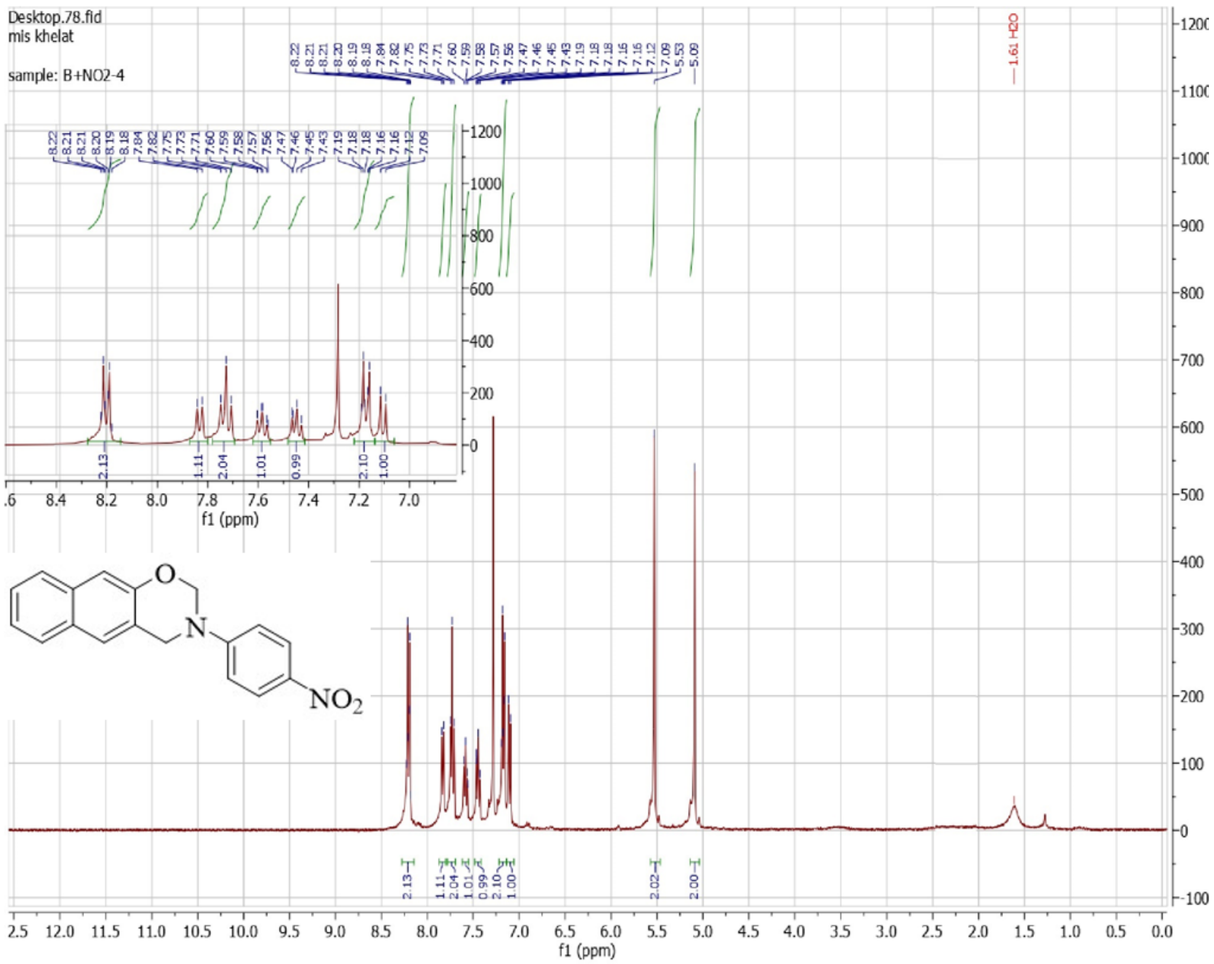

^{1}H NMR of 4f

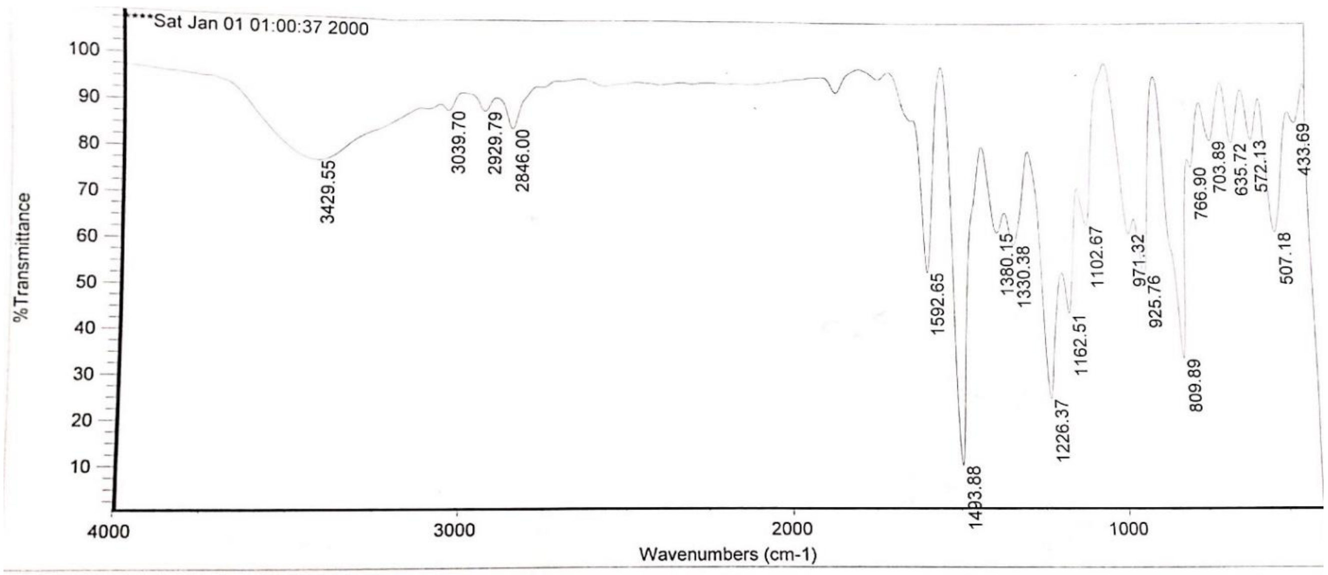

FT-IR of 4g

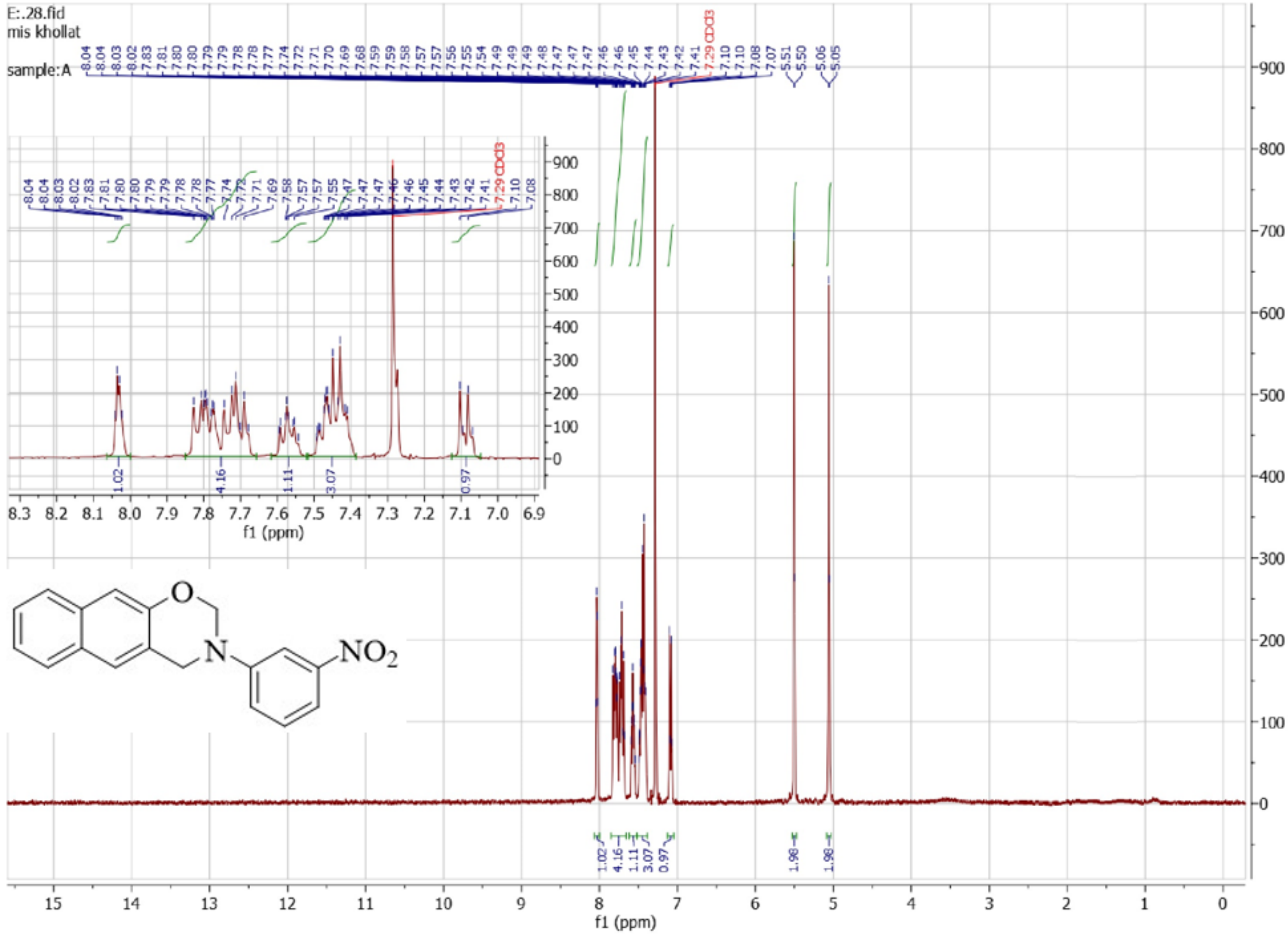

^{1}H NMR of 4g

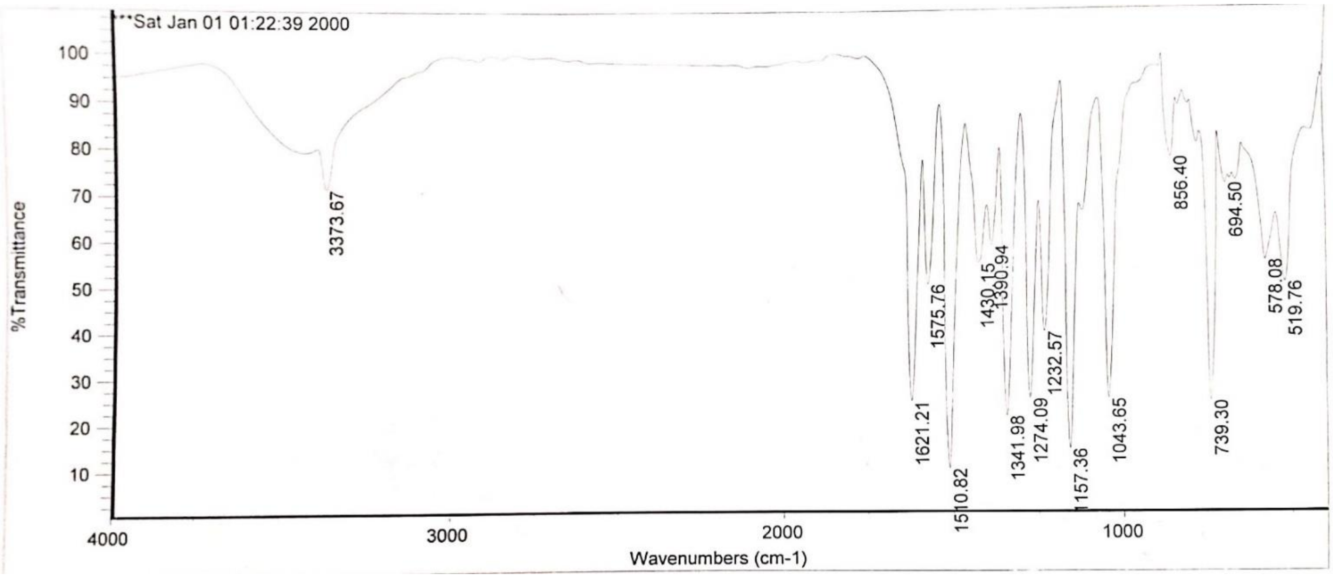

FT-IR of 4h

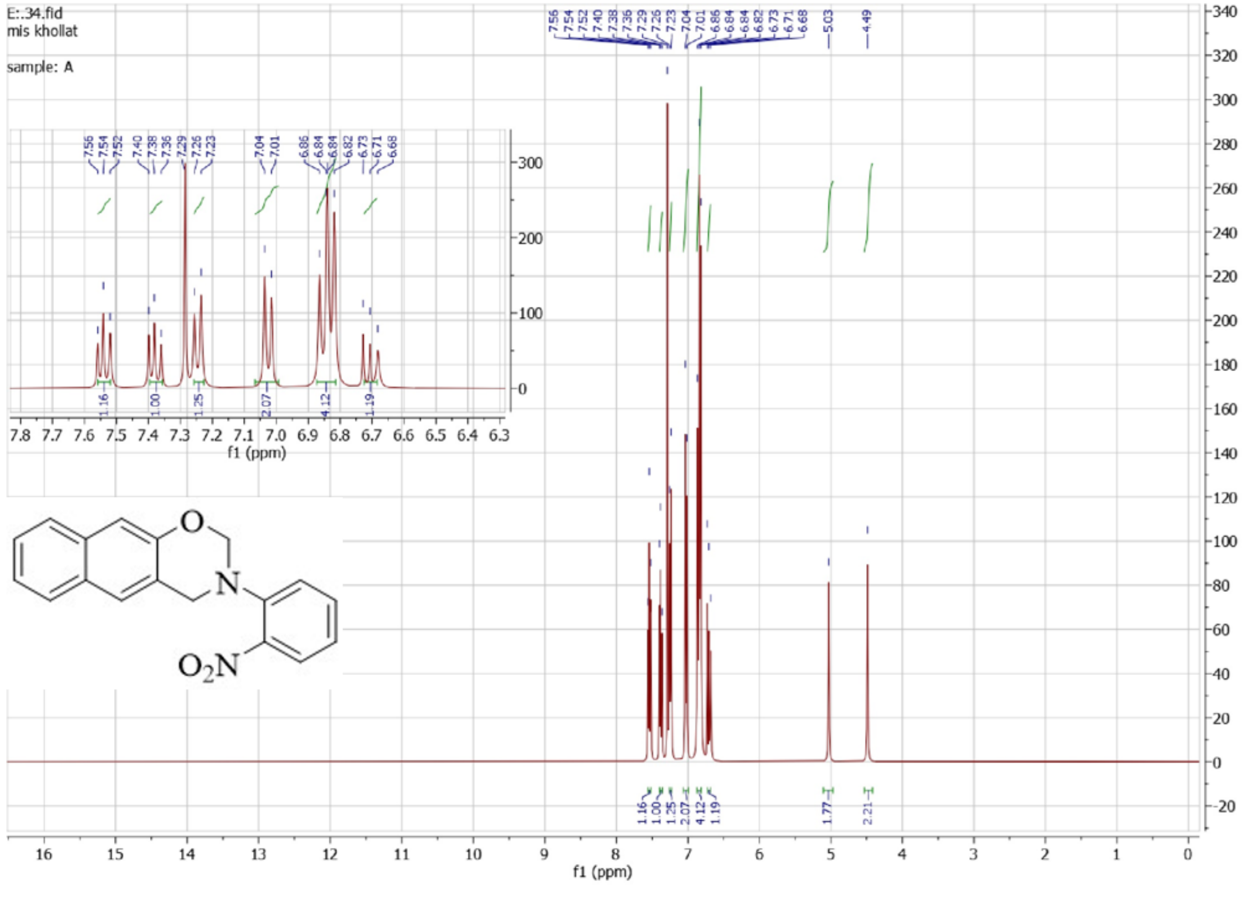

^{1}H NMR of 4h

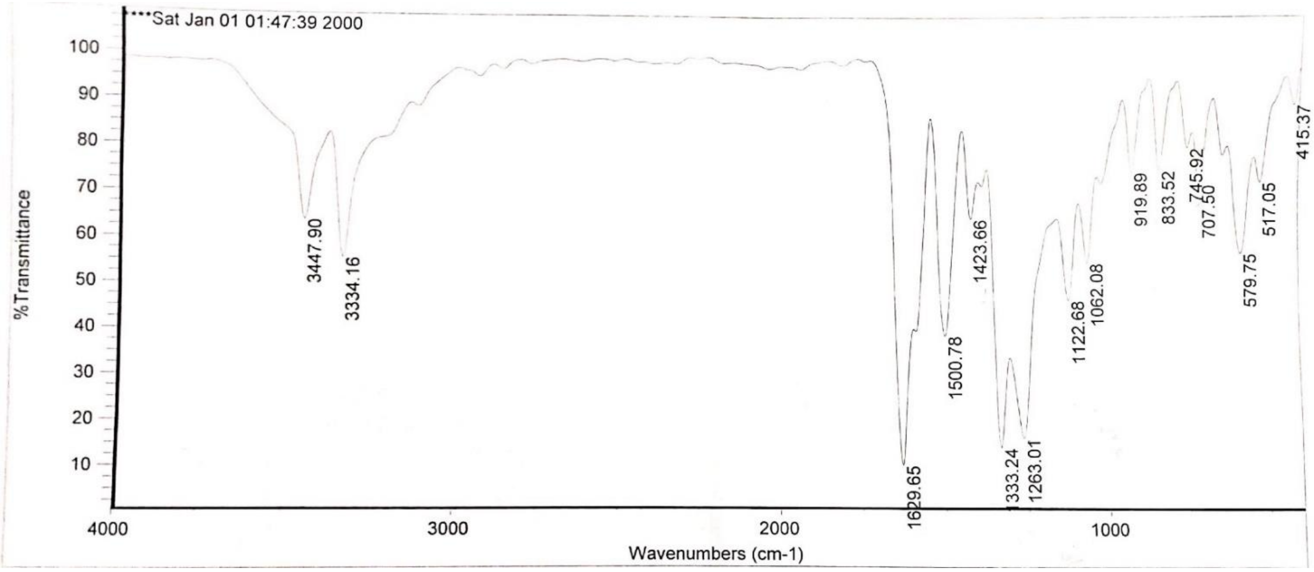

FT-IR of 4i

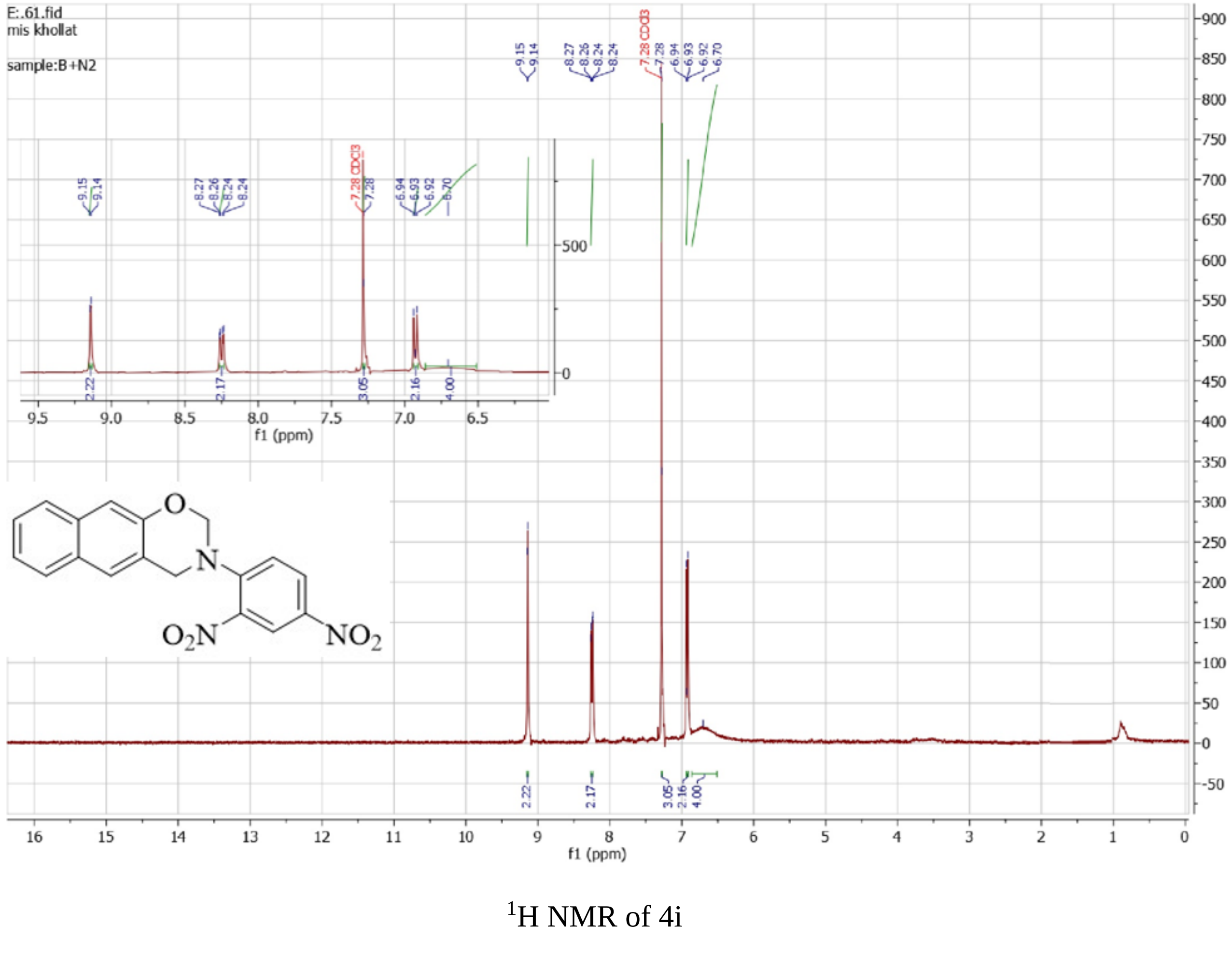

^{1}H NMR of 4i

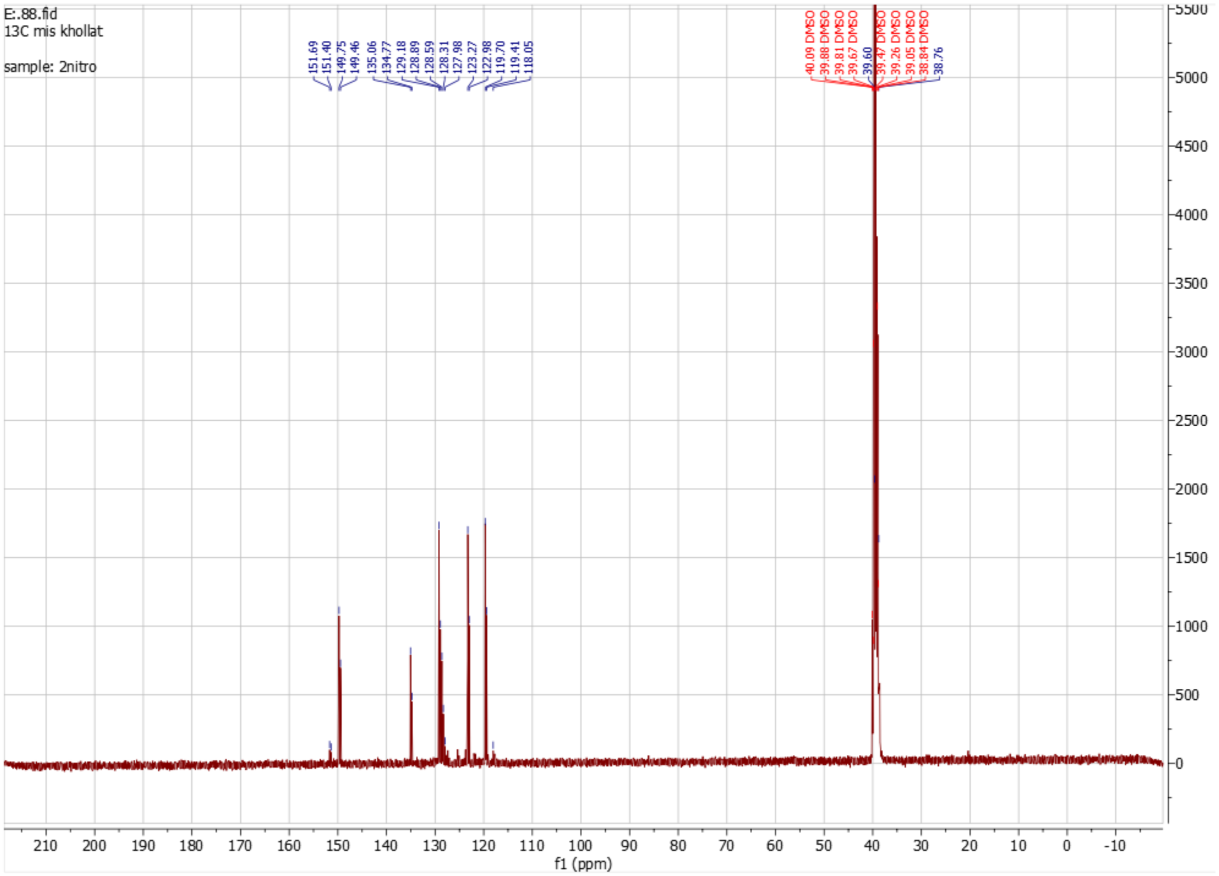

^{13}C NMR of 4i

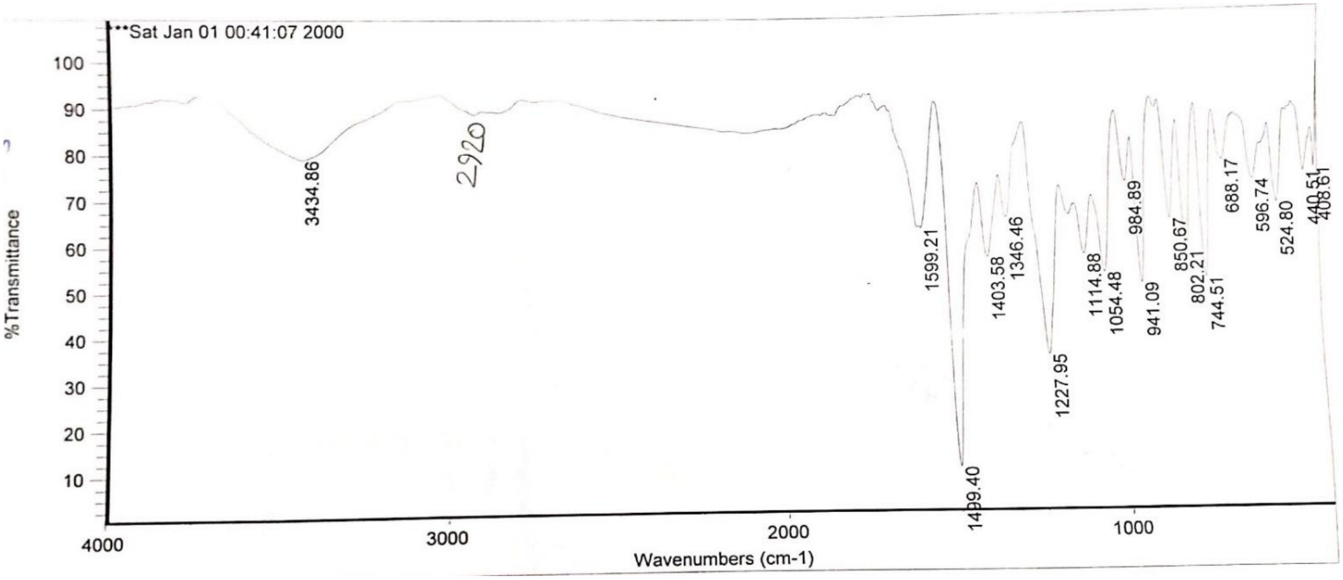

FT-IR of 6a

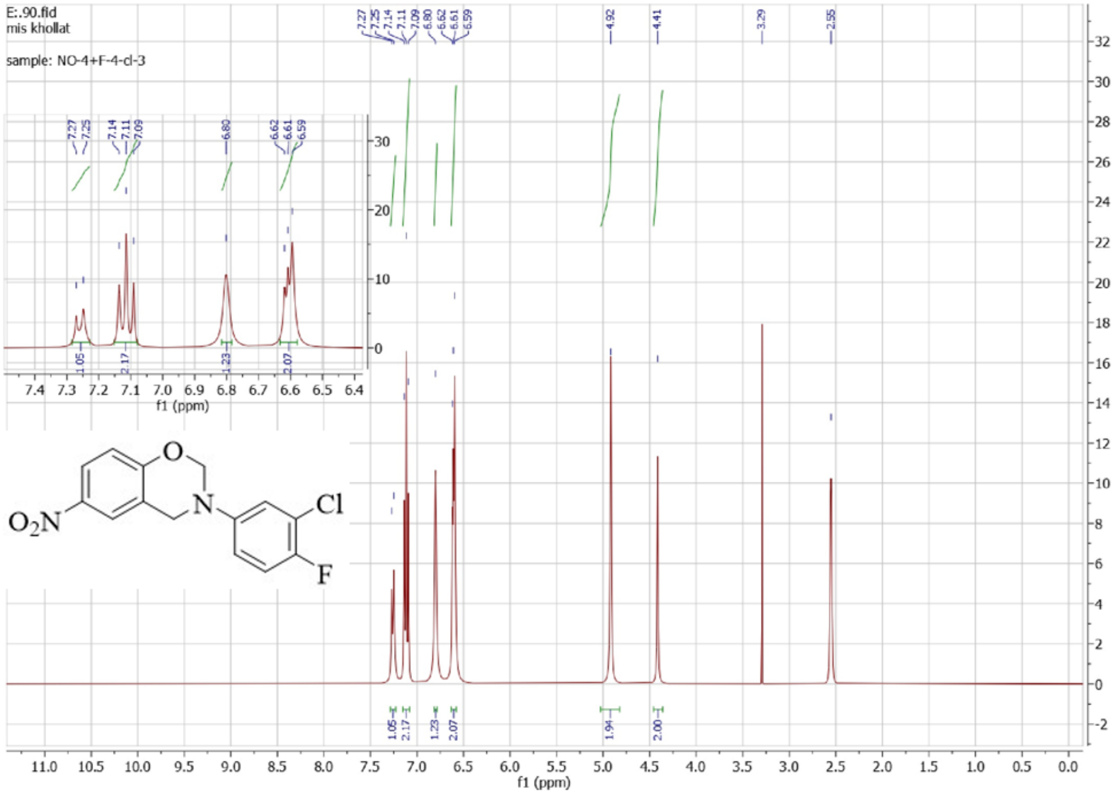

^1H NMR of 6a

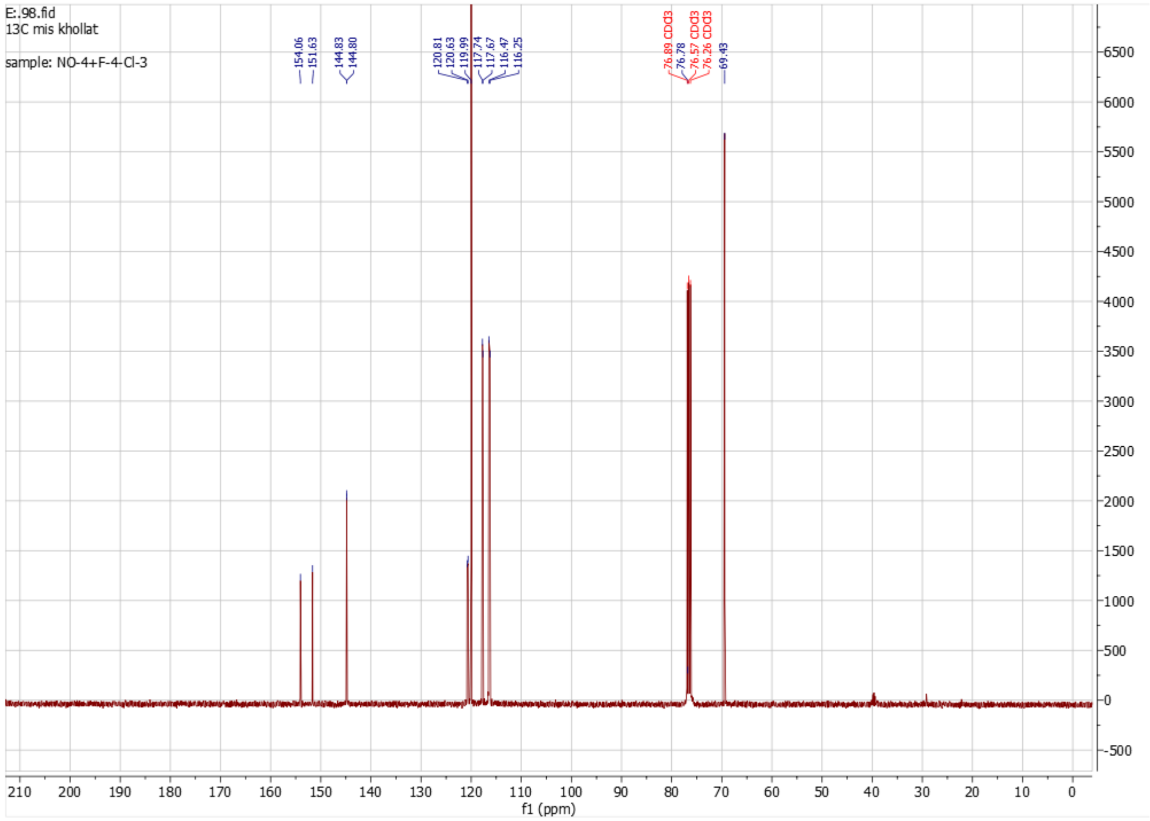

^{13}C NMR of 6a

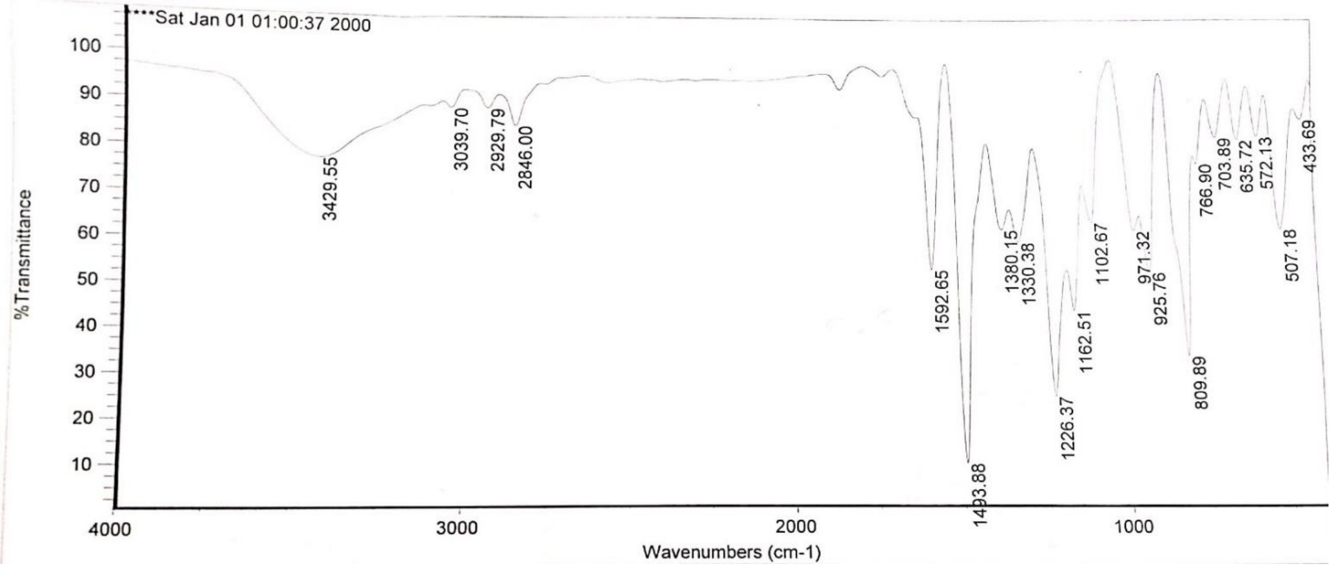

FT-IR of 6b

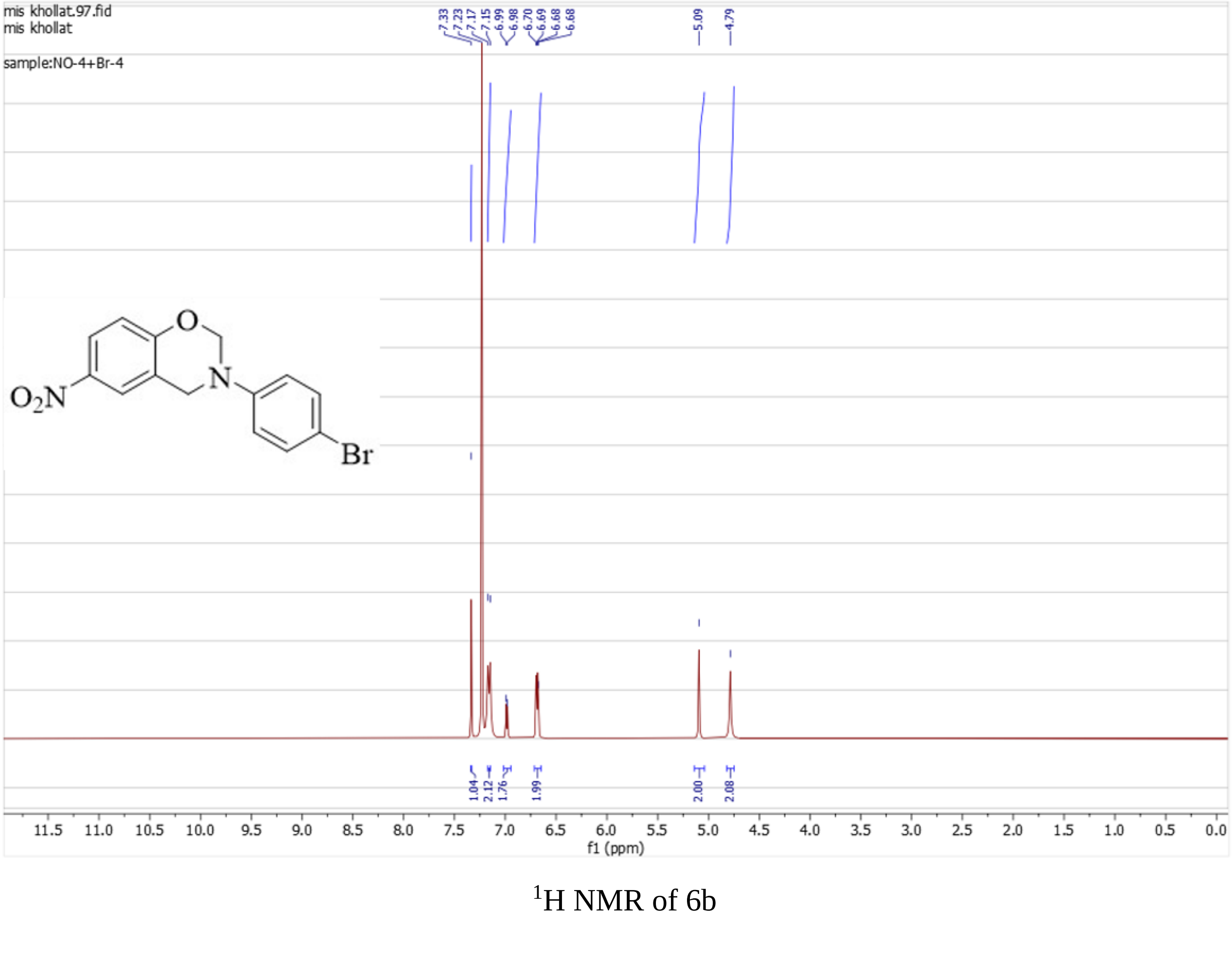

^{1}H NMR of 6b
